# Cross-Modal Alignment and Rectified Flow-Based Latent Representation Synthesis for Enhanced Speech-Driven Alzheimer’s Disease Detection

**DOI:** 10.3390/bioengineering13030370

**Published:** 2026-03-23

**Authors:** Shu Xiang, Haobo Ling, Meihong Wu

**Affiliations:** 1 Department of Artificial Intelligence, Institute of Artificial Intelligence, Xiamen University, Xiamen 361005, China; shuxiang@stu.xmu.edu.cn; 2 School of Informatics, Xiamen University, 422 Siming South Road, Xiamen 361005, China; linghaobo@stu.xmu.edu.cn

**Keywords:** Alzheimer’s disease, EEG, feature alignment, Rectified Flow, latent representation, cross-modal fusion, classification

## Abstract

To address the limited accuracy of speech-based Alzheimer’s Disease (AD) screening and the shortage of paired multimodal data, this paper proposes a detection framework based on feature alignment and Rectified Flow-driven latent representation generation. The EEG dataset consists of 36 AD patients and 29 Healthy Controls (HC). The speech dataset contains 399 samples, which include 114 AD cases, 132 Mild Cognitive Impairment (MCI) cases, and 153 HC cases. We extracted multidimensional features of EEG signals, such as time-domain and frequency-domain characteristics, alongside behavioral representations of speech. A heterogeneous alignment network was used to map these features into a common semantic subspace, where an adaptive interpolation strategy reconstructed the missing pathological trajectories of MCI within the latent space. On this basis, a conditional Rectified Flow model was introduced to learn the optimal transport mapping from speech to EEG. This model generated physiological-information-rich latent representations to compensate for semantic gaps. Experimental results showed that the fused features from speech and latent representations achieved a three-class classification accuracy of 89.08%, a precision of 88.77%, and a recall of 88.71%. This performance represented an accuracy improvement of 9.28% compared with the speech-based baseline system. Our method combines the convenience of speech screening with the high reliability of neurophysiological signals, and it provides a new approach for low-cost early detection of AD.

## 1. Introduction

Alzheimer’s Disease (AD) is an insidious and slowly progressing neurodegenerative disorder, and it is the most common type of dementia. Clinically, it is characterized by progressive memory loss, cognitive decline, and behavioral abnormalities [1]. In its advanced stages, patients lose the ability to perform basic activities of daily living. Healthy Controls (HC) refer to individuals whose cognitive function is at a normal aging level. Mild Cognitive Impairment (MCI) serves as the transitional state between HC and AD, and individuals with MCI represent the group at highest risk for developing AD [2]. Given that the pathological course of AD is irreversible, early diagnosis and intervention are of paramount importance for delaying disease progression and enhancing patients’ quality of life [3,4].

Currently, clinical diagnosis of AD relies heavily on neuropsychological testing, fluid biomarkers, and neuroimaging techniques [5,6,7]. However, these methods face significant limitations. Neuropsychological tests assess cognitive functions via scales. They are easy to administer yet highly subjective and are susceptible to the influences of patients’ educational backgrounds and emotional states [8]. Biomarker assays, such as those involving cerebrospinal fluid, require invasive procedures and complex clinical workflows. Although neuroimaging techniques, including Magnetic Resonance Imaging and Positron Emission Tomography, can directly reveal pathological changes like brain atrophy or protein deposition, they are constrained by high costs, time requirements, and potential radiation risks. Consequently, developing a non-invasive, cost-effective, and accurate diagnostic tool for primary screening has become an urgent priority.

Speech signals and EEG have emerged as vital research carriers in AD diagnosis due to their rich pathological information and the non-invasive, convenient, and cost-effective nature of their acquisition [9]. AD patients exhibit distinct linguistic patterns, including reduced fluency, incoherence, word-finding difficulties, and naming impairments, all of which are reflected in speech signals [10]. EEG captures real-time neural activity and reflects abnormal changes in neuronal firing patterns associated with AD pathology [11]. Analysis methods based on EEG signals have demonstrated outstanding performance in the diagnosis of neurological disorders, such as depression, epileptic focus localization, and sleep disorders. For instance, the adaptive EEG feature engineering framework proposed by Choudhury et al. achieved a binary classification accuracy of 99.22% for depression [12]. Similarly, Chen et al. effectively improved EEG classification performance for epileptic focus localization and deep sleep detection through a lightweight data augmentation strategy [13]. These studies provide valuable methodological references for the application of EEG signals in the diagnosis of neurodegenerative diseases. Concurrently, the rapid advancement of deep learning has provided powerful tools for analyzing these biological signals. Deep learning models possess strong feature learning and data modeling capabilities. They can extract high-dimensional features from raw speech and EEG signals, thereby effectively improving the accuracy and efficiency of AD detection.

Early studies relied predominantly on spatio-temporal statistical features of EEG and machine learning classifiers. However, limited by feature expressivity, performance often hit a bottleneck, with accuracies frequently falling below 80% [14]. Recent studies have demonstrated that processing deep wavelet features or time–frequency image patterns with Convolutional Neural Networks (CNNs), such as ResNet and MobileNet, can improve the detection accuracy to over 97%, which is significantly superior to traditional algorithms [15,16]. Nevertheless, despite the maturity of EEG technology, its reliance on specialized equipment and controlled environments makes it difficult to implement in large-scale community screening. Given that AD patients often exhibit obvious language abnormalities and that speech acquisition is naturally low-cost and portable, analyzing speech signals offers a potentially superior solution for widespread diagnosis.

As early as 2019, researchers analyzed speech features such as pauses and repetitions and achieved an accuracy of 78.3% in automatic dementia recognition using a three-layer neural network [17]. In 2023, Liu et al. proposed a pause feature extraction and encoding strategy based on speech activity detection and achieved an accuracy of 65.4% in AD detection using only acoustic features on the public dataset ADReSS [18]. Although the extraction of speech acoustic features is relatively mature, the information it provides is limited; thus, it is difficult to achieve higher accuracy in AD detection relying solely on speech signals. Textual information, acting as the semantic carrier of speech, plays an irreplaceable role in diagnosis. In 2025, Zhang et al. shifted toward deep semantic mining and proposed the Corrected Automatic Speech Recognition (ASR) Projection (CAP) model [19]. This study constructed dual feature spaces by combining automatic transcription and manual text, introduced four large language models to extract high-dimensional semantic features, and finally achieved an AD detection accuracy of 74.03% on the Chinese dataset NCMMSC 2021 using a bidirectional long short-term memory network classifier.

EEG signals directly reflect neurophysiology and provide high accuracy in AD detection, but their acquisition is costly and complex. Conversely, speech signals are accessible and inexpensive, but their classification performance faces a bottleneck. More importantly, in practical clinical screening, obtaining paired and synchronized EEG and speech data from the same subject is extremely challenging, which directly limits the application of traditional multimodal fusion methods. To address the conflict between modal complementarity and the lack of paired data, this paper proposes a cross-modal enhancement framework based on the Rectified Flow generative model. This framework leverages prior knowledge from high-cost modalities to enhance the representation of low-cost modalities, which enables high-precision AD detection using only speech signals. The main contributions of this paper are as follows:

1. We design an alignment network based on adversarial and metric constraints to map heterogeneous EEG and speech features into a common latent space. This maximizes inter-modal correlation while preserving semantic consistency, and lays the foundation for cross-modal generation.
2. We utilize Rectified Flow to construct a deterministic transport path from speech to EEG features and achieve the generation of high-quality latent representations. This effectively fills the neurophysiological information gap inherent in unimodal speech signal.
3. We develop a dual-stream fusion network incorporating a channel attention mechanism to dynamically integrate speech and generated latent representations. This significantly improves AD detection accuracy while maintaining the convenience of speech-based acquisition.

The organizational structure of this paper is as follows: Section 1 elaborates on the research background, reviews related research progress in the field of AD detection, and clarifies the core innovations of this study. Section 2 details the basic information of the raw EEG and speech datasets. It also explains the construction methods for the relevant experimental data. Section 3 systematically elaborates on the extraction and dimensionality reduction strategies for EEG and speech features. Furthermore, it details the specific implementation methods for cross-modal feature alignment, latent representation generation, and feature fusion. Section 4 presents the various experimental parameter settings of this study and shows detailed experimental results. It also provides an in-depth analysis of the underlying mechanisms behind these results. Finally, Section 5 and Section 6 summarize the key findings of this study, analyze the uncertainties and limitations of the research, and outline prospects for future research directions.

## 2. Materials

High-quality datasets form the foundation for robust model performance and reliable conclusions. Building upon open-source EEG and speech datasets, this study generates EEG image data and text data to fully utilize the original resources. This chapter details the raw data used in the experiments alongside the data preprocessing pipeline. Specifically, it first elaborates on the collection protocols, preprocessing steps, and Power Spectral Density (PSD) map generation strategies for EEG data. Subsequently, it introduces the composition of the speech dataset and the text transcription methods. These procedures provide essential data support for subsequent feature engineering and model training.

### 2.1. EEG Data Preprocessing

EEG signals reflect the real-time activity of brain neurons, and their rhythmic abnormalities serve as critical biomarkers for AD. Because raw EEG signals have high dimensionality and are susceptible to artifact contamination, they require rigorous preprocessing before they can be used for model training.

#### 2.1.1. EEG Dataset

The EEG signals utilized in this study were obtained from a subset of the EEG dataset provided by Miltiadous et al. [20]. The dataset comprises resting-state eyes-closed EEG recordings from 65 subjects, including 36 patients with Alzheimer’s Disease (AD group) and 29 Healthy Controls (HC group) [20]. The cognitive status of all subjects was assessed using the Mini-Mental State Examination (MMSE) scale, where lower scores indicate more severe cognitive decline. The mean MMSE score for the AD group was 17.75±4.5, while the mean score for the HC group was 30. The average age was 66.4±7.9 years for the AD group and 67.9±5.4 years for the HC group [20].

Data acquisition was conducted using a Nihon Kohden 2100 clinical EEG device. According to the international 10–20 system, 19 electrodes (Fp1, Fp2, F7, F3, Fz, F4, F8, T3, C3, Cz, C4, T4, T5, P3, Pz, P4, T6, O1, and O2) and two reference electrodes (A1 and A2) were placed on the scalp [20]. EEG examinations were performed at a sampling rate of 500 Hz and a resolution of 10 μV/mm [20]. All recordings were taken while participants were resting with their eyes closed to minimize external interference. Strict quality control measures were implemented during data collection to ensure accuracy and consistency.

The data preprocessing workflow was as follows. First, a Butterworth band-pass filter (0.5–45 Hz) was applied to remove interference from non-target frequencies, and the signals were re-referenced to the average of A1–A2 channels [20]. Artifact Subspace Reconstruction via the EEGLAB platform was then employed to eliminate artifacts. The duration of the time segment was set to 0.5 s, and any signal whose standard deviation of variation within this time segment exceeded the threshold of 17 was determined as an artifact [20]. A 0.5 s sliding window is the official default setting for the Artifact Subspace Reconstruction technique in the EEGLAB platform. This configuration has undergone extensive validation [21,22] and represents an industry standard for automated EEG artifact detection. An excessively short window length will misclassify physiological transients inherent to EEG signals as artifacts. This error causes an over-rejection of valid data, and it specifically destroys the already weak rhythmic features in the EEG of AD patients. Conversely, an overly long window length reduces the temporal resolution for transient artifacts such as eye movements. Such a setting might mix artifacts with normal EEG signals during evaluation, and this mixture ultimately degrades the precision of artifact removal [23]. In the original dataset, involuntary eye movement artifacts remain present even within eyes-closed resting-state recordings. A 0.5 s window can capture these short-term transient interferences more precisely. Finally, Independent Component Analysis was used to transform the 19 EEG signals into 19 independent components, each representing a distinct neural signal source [20]. This study used datasets that have already been preprocessed by the original authors.

#### 2.1.2. Image Generation Strategy

EEG signals can be categorized into five primary rhythms based on frequency: delta (0.5–4 Hz), theta (4–8 Hz), alpha (8–13 Hz), beta (13–30 Hz), and gamma (>30 Hz) [20]. Further dividing the beta band into beta1 (13–20 Hz) and beta2 (21–32 Hz) allows for a more distinct observation of spectral power differences between AD patients and healthy controls. The power field of beta1 is primarily located in the posterior regions of the brain topographic map, whereas beta2 is largely confined to the prefrontal regions [24]. Previous research studied the spatial distribution of mean power across five frequency bands and found that AD patients exhibit significant power enhancement in low-frequency bands and power suppression in high-frequency bands [25]. Inspired by these findings, this study focused on the 2D topographic mapping of EEG PSD to reveal abnormal distribution of electrophysiological activity.

**Figure 1:**
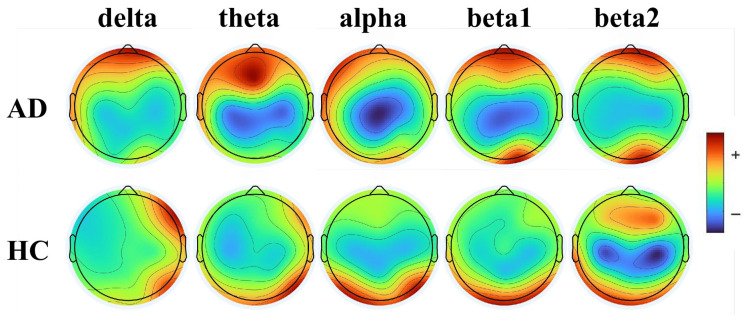
The red color stands for high spectral power and blue for low spectral power. The columns represent five distinct frequency bands, while the rows represent different subject groups (Alzheimer’s Disease (AD) patients and Healthy Controls (HC)).

PSD topographic maps provide a visual representation of the spatial energy distribution of EEG activity at specific frequencies, which is valuable for identifying abnormal brain function patterns. In this study, the Plot Channel Spectra and Maps function of the EEGLAB toolbox was used to construct the EEG image dataset. To effectively reduce dimensionality while capturing the core features, we selected representative center frequency points rather than full-band integration: 2 Hz for delta, 6 Hz for theta, 10 Hz for alpha, 15 Hz for beta1, and 25 Hz for beta2. Through this mapping, raw EEG data for each subject were converted into a sequence of five key PSD topographic maps, as shown in Figure 1.

The PSD topographic maps clearly reveal the slowing characteristic of EEG rhythms in AD patients. In the low-frequency bands (delta and theta), the AD group shows power enhancement (red highlighted areas), which suggests abnormally active slow-wave oscillations. Conversely, the alpha band exhibits marked power suppression (blue low-value areas) and reflects the decline in cortical function. In contrast, the spectral energy distribution in the HC group is more balanced, with no abnormal extremes in specific bands. This spectral shift phenomenon characterized by increased low-frequency components and decreased high-frequency components constitutes a statistically significant electrophysiological difference for distinguishing between AD and HC.

### 2.2. Speech Data and Transcription

Language impairment is one of the core clinical manifestations in AD patients, and speech signals contain rich acoustic and linguistic pathological information. The analysis of speech data relies on acoustic feature extraction from the raw audio alongside the parsing of its corresponding textual content. This subsection introduces the source of the raw speech dataset and elaborates on the specific pipeline for text transcription via ASR technology.

#### 2.2.1. Speech Dataset

This study utilized the official speech dataset provided by the 16th National Conference on Man-Machine Speech Communication (NCMMSC 2021) Alzheimer’s Disease Recognition Challenge. The dataset contains Mandarin speech segments from patients with AD, MCI, and HC [26]. This research specifically selected the long audio samples, which include recordings of subjects performing picture descriptions, fluency naming tasks, and partial free conversations. The training set comprises 280 samples, and the test set includes 119 samples. The sample distribution is shown in Table 1.

#### 2.2.2. Text Transcription

To improve the accuracy of ASR model, speech enhancement was first performed to eliminate environmental noise and improve vocal clarity. We applied a sequence of pre-emphasis, notch filtering, spectral subtraction, and Wiener filtering to suppress power-line interference and steady-state background noise. Subsequently, a band-pass filter and a spectral gain algorithm based on time–frequency signal-to-noise ratio (SNR) were utilized to enhance the harmonic structure of the speech. These procedures significantly improved the SNR and emphasized the clarity of the speaker’s voice.

The FunASR framework was employed to implement the full-link processing from raw speech to structured text [27]. Before recognition, the FSMN-VAD model was used to segment valid speech fragments, which ensured that background noise and long silences did not interfere with subsequent steps. A Paraformer-based non-autoregressive acoustic model then converted acoustic signals into character sequences. Finally, the CT-Transformer model was used to insert punctuation, thereby restoring the readability and syntactic structure of the text. This pipeline improves the robustness and semantic integrity of transcribing non-fluent Mandarin speech. For ground-truth transcriptions, the Praat platform was used for manual labeling, which was further verified through cross-validation by experts with diverse dialect backgrounds to ensure maximum semantic accuracy [28].

## 3. Methodology

To address the heterogeneity of EEG and speech signals and the challenge of insufficient robustness in unimodal diagnosis, this study proposes a cross-modal fusion diagnostic framework based on feature alignment and flow-model-driven generation. This chapter systematically elaborates on the multidimensional feature extraction pipeline, alongside feature alignment and generation strategies. Finally, it introduces the feature fusion and classification methods to establish the mapping from raw signals to AD diagnostic results.

### 3.1. Framework Overview

The framework concurrently extracts time–frequency and nonlinear functional representations from EEG signals, along with acoustic and linguistic features from speech signals, which forms multidimensional heterogeneous input. Subsequently, an alignment network incorporating metric constraints and domain-adversarial mechanisms maps these heterogeneous features into a modality-invariant common semantic subspace. An adaptive interpolation strategy is introduced within this latent space to reconstruct the missing MCI pathological trajectories. Based on this, the core generation module utilizes a conditional Rectified Flow to learn the deterministic optimal transport path from speech to EEG features, where speech signals explicitly guide the generation of latent representations rich in neurophysiological priors. Finally, a fusion module integrates speech features with the generated latent representations via an attention mechanism to achieve high-precision and robust classification of AD, MCI and HC.

### 3.2. Multidimensional Feature Engineering

Effective feature representation is a prerequisite for achieving high-precision diagnosis. Because the physical properties of EEG and speech differ vastly, distinct feature extraction strategies must be adopted. This subsection details the multi-domain feature extraction schemes for EEG signals alongside the dual-dimensional (acoustic and linguistic) extraction schemes for speech signals. Furthermore, it elaborates on the dimensionality reduction and reorganization strategies for high-dimensional features to construct unimodal feature sets with high discriminative power.

#### 3.2.1. EEG Feature Extraction

The extracted EEG features encompass the time domain, frequency domain, time–frequency domain, multiscale entropy (MSE), functional connectivity, and deep spatial features extracted via MobileNetV2.

Time-domain statistical features include the mean, peak, variance, standard deviation, skewness, kurtosis, and root mean square (RMS) [29]. These seven features are stacked and flattened across all channels to construct the input vector. For each subject, the dimension of the EEG time-domain feature vector is 133, structured as

(1) $$v=[\underset{\mathrm{Means}}{\underset{︸}{\mu_{1},\mu_{2},\ldots ,\mu_{19}}},\underset{\mathrm{Peaks}}{\underset{︸}{P_{1},P_{2},\ldots ,P_{19}}},\ldots ,\underset{\mathrm{RMS}\;\mathrm{values}}{\underset{︸}{{\mathrm{RMS}}_{1},{\mathrm{RMS}}_{2},\ldots ,{\mathrm{RMS}}_{19}}}],$$

where v denotes the time-domain feature vector, μ represents the mean value, *P* denotes the peak value, and RMS is the abbreviation for root mean square. To ensure training stability and convergence, these vectors are normalized using Z-Score standardization [30].

Frequency-domain features include relative power, frequency band ratios, median frequency, and spectral entropy. EEG spectra are divided into five bands: delta (0.5–4 Hz), theta (4–8 Hz), alpha (8–13 Hz), beta (13–30 Hz), and gamma (30–100 Hz). Each frequency band’s absolute power should be calculated first. Relative power is defined as the ratio of the absolute power of a specific band to the total power across all five bands. Studies have shown that AD patients exhibit lower spectral power in the alpha band and higher spectral power in the delta band, which is significantly different from that in the healthy elderly population [31]. The three frequency band ratios are the Theta-to-Alpha absolute power ratio, the Delta-to-Alpha ratio, and the comprehensive energy ratio, which is defined as the ratio of low-frequency energy (theta and delta) to high-frequency energy (alpha and beta). The median frequency refers to the frequency point that divides the power spectrum energy into two equal parts and can reflect the shift of the spectral center of gravity. Spectral entropy is calculated to quantify the complexity and disorder of the power spectrum distribution. In its calculation process, the PSD of the signal is first normalized to construct a relative power probability distribution at discrete frequency points. Then, the Shannon entropy is calculated based on this probability distribution and standardized using the theoretical maximum entropy. Finally, the normalized spectral entropy is obtained. In total, each EEG channel has 10-dimensional frequency-domain features. The concatenation and standardization methods are consistent with those for time-domain features. The dimension of the final frequency-domain feature is 190.

Time–frequency features are quantified using the Discrete Wavelet Transform (DWT) with the Daubechies 4 (db4) mother wavelet and a 5-level decomposition to obtain six sub-bands (A5, D1–D5). The energy, standard deviation, and Shannon entropy are calculated for each sub-band. The dimension of the final time–frequency feature is 342.

Traditional single-scale entropy analysis only focuses on signal irregularities at a single time scale, which easily overlooks the complex structures of biological systems across multiple temporal and spatial scales. Therefore, this study introduces MSE features to quantify the nonlinear characteristics of EEG signals [11,32]. Entropy continues to decrease in AD patients across most scales [33]. This algorithm first performs multiscale coarse-graining on the downsampled signal. For a discrete time series *x* with length *N*, the coarse-grained sequence $y^{\left(\tau \right)}$ at the scale factor τ is obtained by calculating the arithmetic mean of data points within non-overlapping windows. For the scale τ (1≤τ≤20), the j-th coarse-grained data point $y_{j}^{\left(\tau \right)}$ is calculated as

(2) $$y_{j}^{\left(\tau \right)}=\frac{1}{\tau }\sum_{i=(j-1)\tau +1}^{j\tau }x_{i},\;1\leq j\leq \left\lfloor N/\tau \right\rfloor .$$

Sample entropy is then calculated for each coarse-grained sequence:

(3) $$\mathrm{SampEn}\left(m,r\right)=-\ln\frac{A}{B},$$

where SampEn(m,r) denotes the sample entropy; *m* represents the embedding dimension; *r* is the similarity tolerance; ln is the natural logarithm function; *A* refers to the number of pairs of *m*-dimensional vectors that have a Chebyshev distance less than *r* after adding one more dimension; *B* denotes the number of pairs of *m*-dimensional vectors that have a Chebyshev distance less than *r*. Based on the empirical values of EEG analysis, we set the embedding dimension *m* as 2 and the similarity tolerance *r* as 0.15 times the standard deviation of the original data of the current channel. This study calculates the sample entropy values across scales 1 to 20 for each of the 19 channels and concatenates the features of all channels to form the final MSE feature vector with a dimension of 380.

We employ the Phase Lag Index (PLI) to quantify the synchronization degree of neural activities between different brain regions and construct functional connectivity features. First, FIR filters are used to extract alpha band signals, and the Hilbert transform is applied to obtain the instantaneous phase ϕ(t) of each channel. For channels i and j, the PLI value of their phase difference is calculated as

(4) $${\mathrm{PLI}}_{i,j}=\left|\langle \mathrm{sign}\left(\sin\left(\Delta \phi_{i,j}\left(t\right)\right)\right)\rangle \right|.$$

In this equation, ${\mathrm{PLI}}_{i,j}$ is the Phase Lag Index between channel *i* and channel *j*. Here, *i* and *j* are EEG channel indices, $\Delta \phi_{i,j}\left(t\right)$ is the instantaneous phase difference between the two channels at time *t*, sign(·) is the sign function, sin(·) is the sine function, 〈·〉 indicates time averaging, and |·| is the absolute value operator. The brain is divided into five regions of interest (ROIs): Frontal, Central, Temporal, Parietal, and Occipital. Connectivity strength is defined as the mean PLI value across all electrode pairs between two ROIs. Ultimately, by calculating all pairwise regional combinations, a functional connectivity feature vector containing 10 components is constructed.

**Figure 2:**
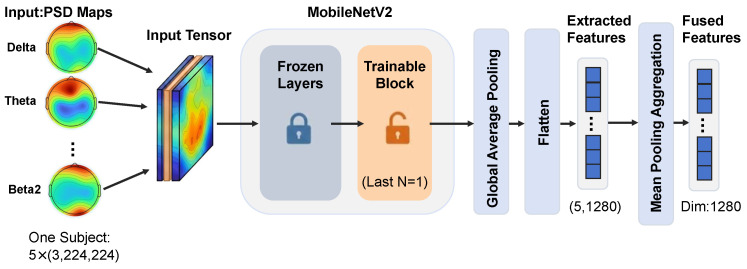
Power Spectral Density (PSD) deep feature extraction flowchart.

To capture high-dimensional spatial topological features from multi-band PSD topographic maps, this study selects MobileNetV2, a lightweight CNN, as the feature extractor [34]. Compared with traditional deep networks, its parameter-efficient architectural design is more suitable for medical datasets with limited sample sizes, which can effectively mitigate the risk of overfitting while accurately extracting the textural features of energy distribution across brain regions. The MobileNetV2 backbone network adopted in this study is loaded with pre-trained weights on the ImageNet-1k dataset, and a layer-wise fine-tuning strategy is employed. Most convolutional layer parameters in the front segment are frozen, only the terminal convolutional blocks are unfrozen, and parameter update and adaptation are performed using PSD topographic maps. The following operations are implemented for multi-band input data. First, the 5 PSD images of each subject are reshaped into independent samples and input into the backbone network in parallel. After multiple layers of convolution and downsampling, a deep feature vector with a dimension of 1280 is output through the global average pooling layer. Then, the model performs mean aggregation on the 5 frequency-band feature vectors of the same subject to generate a unique representation. The PSD image feature extraction process is shown in Figure 2.

#### 3.2.2. Speech Acoustic and Linguistic Features

This section extracts multidimensional audio features. Acoustic representations include the ComParE paralinguistic feature set, deep features from Mel-spectrograms, and HuBERT-based speech representations [35]. Textual representations combine custom lexical features, Term Frequency–Inverse Document Frequency (TF-IDF) statistical features, and BERT-based semantic features.

The OpenSMILE toolbox is utilized to extract the ComParE feature set, which is built upon 38 Low-Level Descriptors (LLDs) covering prosody, spectrum, and voice quality. To capture the temporal dynamics of the speech signal, the first- and second-order derivatives of these LLDs are calculated, and then 21 statistical functionals are applied for global aggregation. This process maps the raw speech signal to a 6373-dimensional feature vector and integrates frame-level acoustic details.

**Figure 3:**
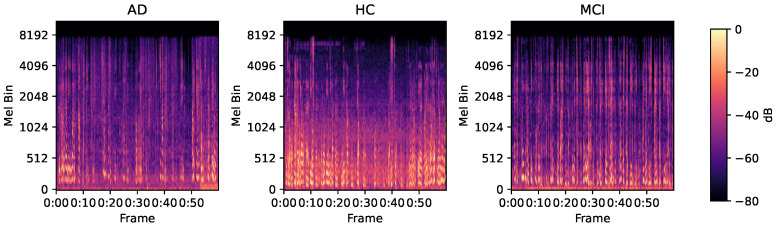
In a Mel spectrogram, bright color represents the subject’s vowel formants and fundamental frequency energy, with their continuity reflecting the coherence of speech; dark colors corresponds to speech gaps or silent segments. In the spectrograms of AD patients, the dark areas are significantly increased, which indicates pathological features such as prolonged pauses and decreased articulation clarity. In healthy individuals, the bright stripes are closely arranged, which means they have coherent speech and richer emotional expression. The spectrograms of Mild Cognitive Impairment (MCI) patients fall somewhere in between the two.

A Mel spectrogram can convert a one-dimensional time-domain speech signal into a two-dimensional time–frequency energy map that aligns with the nonlinear auditory perception of the human ear [36]. This study extracts Mel spectrogram features based on the Librosa library. Audio is resampled to 22,050 Hz and segmented into two segments to ensure feature completeness. The process involves a Short-Time Fourier Transform (STFT), mapping the linear power spectrum to a Mel scale by non-uniform triangular filters and applying a logarithmic transform to enhance low-energy details. All spectrograms are standardized to (128, 2584), which represents the number of Mel bands and the number of time frames, respectively. The Mel spectrograms of patients with AD, healthy controls, and MCI are shown in Figure 3.

**Figure 4:**
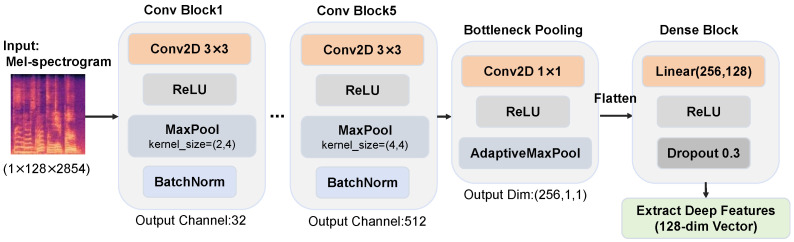
The Convolutional Neural Network (CNN) architecture for Mel spectrogram feature extraction.

A five-layer 2DCNN is designed to extract deep acoustic features from these spectrograms. The backbone consists of five convolutional modules with filter counts of 32, 64, 128, 256, and 512. Considering that the Mel spectrogram spans a much larger range on the time axis than on the frequency axis, this model designs a specific asymmetric max-pooling kernel in order to significantly compress the time dimension while preserving frequency resolution. For 128-dimensional frequency band inputs, the pooling kernel sizes are set sequentially as [(2,4), (4,4), (2,5), (2,4), (4,4)]. This design results in a much higher downsampling rate in the time dimension than in the frequency dimension, which effectively addresses the dimensionality problem of long speech inputs. The CNN architecture for Mel spectrogram feature extraction is shown in Figure 4.

This study uses the HuBERT model as a self-supervised feature extractor to obtain context representations that are highly sensitive to speaker style, prosody, and content [35]. Since the dataset consists of Chinese speech, to maximize adaptation to the linguistic characteristics, the experiment selects the TencentGameMate/chinese-hubert-large model, which has been fine-tuned on a large Chinese corpus [37]. This model contains 24 Transformer encoder layers with a hidden dimension of 1024. The feature acquisition process is as follows: First, all input audio signals are resampled to 16 kHz, which is the standard sampling rate used in the model pre-training phase, and then waveform normalization is performed. The preprocessed waveforms are fed into the HuBERT model, with the output of the last hidden layer extracted as the frame-level feature sequence. Finally, average pooling is performed on the frame-level feature sequence along the temporal dimension, and the local features are aggregated into a 1024-dimensional global feature vector, which serves as the acoustic representation of the subject.

The following is the process for linguistic feature extraction. In the early stage, patients with AD often exhibit symptoms such as lexical retrieval deficits and memory decline, which are specifically manifested as reduced vocabulary size, word repetition, and excessive use of pronouns [38]. To quantify such changes, this study extracts two categories of lexical features, namely lexical diversity and part-of-speech distribution features, from transcribed texts. Lexical diversity is used to evaluate the richness of subjects’ vocabulary, which is quantified by the Type–Token Ratio, Brunét’s Index, and Honoré’s Statistic. For part-of-speech distribution features, the Jieba word segmentation tool is used to perform part-of-speech tagging on the cleaned text, followed by the separate calculation of the Pronoun-to-Noun Ratio (PNR) and content word density. PNR is defined as the ratio of the number of pronouns to that of nouns. Content word density is calculated by the noun ratio and verb ratio respectively. The language of AD patients tends to be empty descriptions, characterized by a decrease in the proportion of content words and an increase in the proportion of function words. These six features are concatenated to form a 6-dimensional lexical feature vector.

This study employs TF-IDF to construct text vectorized representations, which enables the acquisition of statistical differences in word preference among AD patients [39]. First, this experiment utilizes the Jieba word segmentation tool to perform segmentation on transcribed texts in accurate mode and then builds a bag-of-words model based on TfidfVectorizer. Due to the inherent sparsity of small-scale medical datasets, this study does not retain all vocabulary, but limits the capacity of the feature space to the 1000 words with the highest frequency in the entire corpus. The TF-IDF algorithm assigns higher weights to words that are frequently used locally but rare in the global corpus. It can effectively suppress the interference from high-frequency function words and automatically screen out the most discriminative pathological language features.

The BERT model is used to extract semantic features from transcribed texts. As a pre-trained model based on a multi-layer Transformer encoder, BERT can capture long-range contextual dependencies through the self-attention mechanism, thereby generating representations for long texts. The experiment selects bert-base-chinese as the pre-trained model, which consists of 12 Transformer encoder layers with a hidden layer dimension of 768. The BERT model is fine-tuned on our dataset. A linear classification head is added to the output layer, and all weights of the model are updated via the backpropagation algorithm to align its semantic space with specific pathological features. After fine-tuning, the classification head is removed, and the 768-dimensional vector corresponding to the CLS token in the last hidden layer of BERT is extracted. This vector aggregates the global contextual representation of the semantic information of the entire sentence.

#### 3.2.3. Feature Dimensionality Reduction and Reorganization

**Figure 5:**
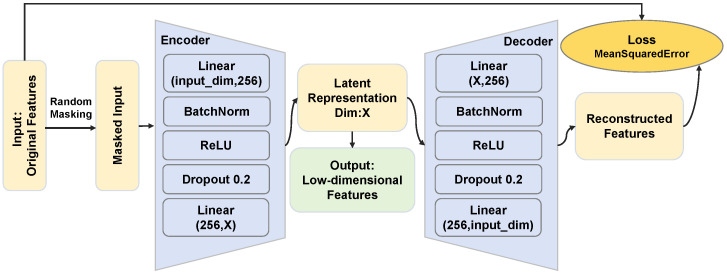
The architecture of Masked Autoencoder (MAE).

Raw EEG and speech features usually have extremely high dimensionality and sparsity. Directly feeding them into subsequent models easily triggers the curse of dimensionality, which weakens the generalization ability of the models. Therefore, this study performs reasonable dimensionality reduction on all features, which refers to Principal Component Analysis (PCA) and Masked Autoencoder (MAE). These two methods respectively proceed from the perspectives of linear denoising and nonlinear high-dimensional feature mining and jointly provide a more discriminative feature foundation for subsequent generative models. Among them, as a classical statistical linear dimensionality reduction method, PCA can efficiently eliminate global redundancy and background noise in data by virtue of its orthogonal transformation property [40]. As a nonlinear method based on self-supervised learning, MAE forces the model to learn the deep latent structure of data through the mask reconstruction mechanism, and it has stronger semantic representation ability when processing complex nonlinear signals. This study adopts a general MAE architecture, where the hidden layer dimension of both the encoder and decoder is set to 256. Dimensionality reduction occurs at the output end of the encoder, with different dimensions set according to different input, and the masking ratio is set to 0.3. The architecture of MAE is shown in Figure 5.

To effectively compress feature dimensionality while preserving the intrinsic structural information of raw EEG signals to the greatest extent, this study adopts the cumulative explained variance ratio as the criterion for selecting the number of principal components. Considering that EEG signals have the characteristics of non-stationarity and contain a certain degree of background noise, an excessively high retention rate may introduce redundant noise, while an excessively low retention rate may lead to the loss of key pathological features. After experimental verification and trade-off, we set the threshold of cumulative explained variance ratio to 95%. For complex nonlinear high-dimensional features such as PSD images, MAE is employed for dimensionality transformation, and the optimal dimensionality is selected based on the classification accuracy of downstream tasks. The dimensionality changes of EEG and speech features are shown in Table 2.

### 3.3. Cross-Modal Mapping and Latent Representation Generation

This subsection first introduces the heterogeneous feature alignment strategy, which is designed to eliminate structural discrepancies between modalities. Subsequently, it details the principles of the Rectified Flow-based generative model. This model is responsible for learning the mapping relationship from the speech feature space to the EEG feature space.

#### 3.3.1. Heterogeneous Feature Alignment Strategy

This study constructs a cross-modal feature alignment network to map speech and EEG features into a modality-invariant latent space through mapping, adversarial training, and metric constraints. To address the asymmetric issue of missing MCI categories in EEG data, we propose an adaptive interpolation strategy based on manifold continuity.

**Figure 6:**
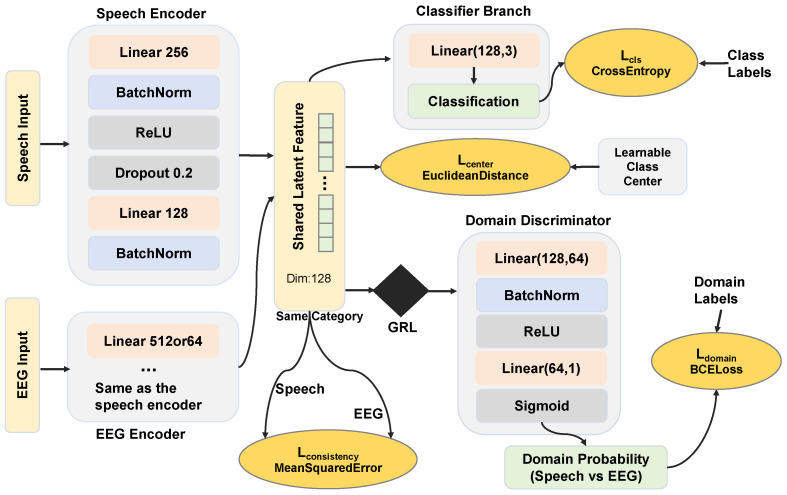
The architecture and loss function settings of the cross-modal feature alignment network.

The core mechanisms of the alignment network include feature mapping, metric constraint, and domain-adversarial alignment. Feature mapping refers to the use of two independent encoders to nonlinearly map speech and EEG features to a subspace of the same dimension. Metric constraint consists of center loss and consistency loss constraint. The center loss forces samples of each category (whether from the speech or the EEG modality) to be closely clustered around the category center in the latent space, so as to reduce the intra-class distance. The consistency loss enforces the geometric coincidence of the speech feature center and the EEG feature center for the same category, thereby achieving cross-modal semantic alignment. Domain-adversarial alignment is implemented by introducing a Gradient Reverse Layer (GRL) and a domain discriminator. The discriminator tries to distinguish which modality the features come from, while the encoders try to fool the discriminator. When the discriminator fails to make a distinction, it indicates that the feature distributions of the two modalities are completely indistinguishable, and the inter-modality differences are eliminated. The model architecture is shown in Figure 6.

The alignment model adopts a multi-task joint optimization strategy, and the total loss function is composed of four weighted components. The classification loss adopts the weighted cross-entropy loss, where the weight coefficients are determined by the reciprocal of the sample proportions of AD, HC, and MCI in the training set to alleviate the gradient bias caused by category imbalance. The center loss is the mean of the Euclidean distances between features and their category centers. To prevent its gradient from dominating the main task, the parameters of this term are updated via an independent SGD optimizer. The domain-adversarial loss introduces a GRL and confuses the modality sources through a binary classifier. The adaptation factor of GRL dynamically increases from 0 to 0.1 during the training process, aiming to prioritize the feature extraction capability in the early stage of training and gradually enhance modality invariance in the later stage. The consistency constraint loss calculates the mean squared error between the mean values of speech and EEG features of the same category, which enforces the geometric alignment of bimodal semantics. For MCI samples, this term is automatically masked and degenerates into a unimodal constraint. The total loss function is calculated as

(5) $$L_{\mathrm{total}}=L_{\mathrm{cls}}+\lambda_{\mathrm{center}}L_{\mathrm{center}}+\lambda_{\mathrm{domain}}L_{\mathrm{domain}}+\lambda_{\mathrm{consist}}L_{\mathrm{consist}}.$$

where $L_{\mathrm{total}}$ is the overall loss for multi-task joint optimization; $L_{\mathrm{cls}}$ is weighted cross-entropy classification loss; $L_{\mathrm{center}}$ is center loss; $L_{\mathrm{domain}}$ is domain-adversarial loss; $L_{\mathrm{consist}}$ is consistency loss; $\lambda_{\mathrm{center}}$, $\lambda_{\mathrm{domain}}$, $\lambda_{\mathrm{consist}}$ are weight coefficients for each loss component. This study conducted a systematic grid search on the validation set to determine the optimal hyperparameter combination. The final optimal weights are determined as 0.05 for $\lambda_{\mathrm{center}}$, 0.1 for $\lambda_{\mathrm{domain}}$, and 5.0 for $\lambda_{\mathrm{consist}}$. The gradient magnitude of the consistency loss calculated by mean squared error is significantly smaller than that of cross-entropy. Therefore, a larger weight is assigned to $\lambda_{\mathrm{consist}}$ to ensure that the encoder receives sufficient geometric alignment gradient signals. This specific value is rigorously validated through parameter sensitivity analysis. The parameter $\lambda_{\mathrm{domain}}$ is set to 0.1 to balance modality confusion and semantic discriminability. This setting prevents an overly strong discriminator from causing the loss of feature semantics. The parameter $\lambda_{\mathrm{center}}$ serves merely as an auxiliary regularization term. It is assigned a smaller weight to prevent excessive collapse of the feature space and to ensure generalization ability.

In the clinical pathology of AD, the internationally recognized Alzheimer’s Continuum hypothesis states that cognitive impairment is not a sudden event. Rather, it is a progressive degenerative process that evolves from cognitively healthy controls, through MCI, and ultimately to dementia [41]. When mapped into the high-dimensional latent space of deep learning, this clinical degenerative process should theoretically manifest as a continuous manifold trajectory pointing from the HC feature cluster to the AD feature cluster. Given the high scarcity of cross-modal paired data (especially high-quality EEG data in the MCI stage) in clinical collections, this study introduces a prior regularization strategy based on latent space manifold interpolation during the model training phase. Specifically, when constructing the common semantic subspace of the alignment network, the algorithm synthesizes anchor features that represent the transitional state. It achieves this by performing linear interpolation in the latent space between the real HC sample features ($z_{\mathrm{HC}}$) and AD sample features ($z_{\mathrm{AD}}$) within the same batch. This process is described as follows:

(6) $$z_{\mathrm{MCI}\_\mathrm{pseudo}}=\alpha z_{\mathrm{AD}}+\left(1-\alpha \right)z_{\mathrm{HC}}.$$

Here, α∈(0,1) is a dynamic mixing coefficient that follows a Beta distribution. It must be strictly stated that the aforementioned manifold interpolation process serves solely as a spatial regularization method during the training phase. Its core objective is to bridge the representational gap between HC and AD in the latent space. This strategy prompts the model to learn smooth decision boundaries that conform to the medical prior of the disease continuum. Furthermore, it provides continuous gradient guidance of pathological evolution for the generative model, which prevents the occurrence of mode collapse during the flow matching process.

#### 3.3.2. Rectified Flow-Based Generative Model

This study constructs a conditional Rectified Flow model based on velocity field estimation, which aims to learn a deterministic ordinary differential equation (ODE) that smoothly transports the standard Gaussian noise distribution to the target EEG feature distribution, and whose generation process is explicitly guided by speech features [42].

The conditional velocity field network is used to fit the vector field required for flow matching. This network receives three sets of input signals, namely the flow state $x_{t}$ at the current moment, the time step t∈[0,1], and the speech feature vector *c* serving as the generation condition. The time step *t* and the condition *c* are respectively projected to a hidden layer dimension of 256 through two independent two-layer fully connected layers with the SiLU activation function. The flow state $x_{t}$ is mapped to the same dimension via a linear projection layer. The three components are then added element-wise to obtain early fusion features. The fused feature vector sequentially passes through 3 stacked residual blocks, where each block consists of two fully connected layers with the SiLU activation function, and layer normalization is introduced to accelerate convergence. At the end of the network, a linear projection layer maps the high-dimensional hidden features back to the original EEG data dimension, and outputs the instantaneous velocity vector at the current moment, which is used to guide the numerical integration process of the ordinary differential equation.

To train the conditional velocity field network to learn the transport trajectory from noise to data, we leverage the Rectified Flow framework to construct an end-to-end generation pipeline. This module has no learnable parameters and is mainly responsible for defining the evolution equation and optimization objective of the probability flow. Rectified Flow assumes that there exists a straight-line transport path between the noise distribution $\pi_{0}$ (standard normal distribution) and the target data distribution $\pi_{1}$ (real EEG features) [42]. For any time step *t* (0≤t≤1), the intermediate flow state $x_{t}$ is explicitly constructed in the form of linear interpolation, which is calculated as

(7) $$x_{t}=t\cdot x_{1}+\left(1-t\right)\cdot x_{0},$$

where $x_{0}$ is the initial noise (standard normal distribution), $x_{1}$ is a real sample, and $x_{t}$ is the intermediate state at time *t*. This construction method ensures that the evolution path from noise to data is the geometrically shortest straight line and alleviates the optimization complexity during model convergence. The training objective of the model is to make the velocity field $v_{\theta }\left(x_{t},t,c\right)$ predicted by the velocity network approximate the theoretical derivative of the aforementioned straight-line path. Since the time derivative of the straight-line path is always $\left(x_{1}-x_{0}\right)$, the loss function is defined as the mean squared error between the predicted velocity and the target velocity, which is calculated as

(8) $$L=E_{t,x_{0},x_{1}}\left[\right|v_{\theta }\left(x_{t},t,c\right)-\left(x_{1}-x_{0}\right){|}_{2}^{2}],$$

where L is the mean squared error loss, E[·] denotes expectation over *t*, $x_{0}$, and $x_{1}$, $v_{\theta }\left(x_{t},t,c\right)$ is the velocity field predicted by the network (parameterized by θ, with conditional information *c*), $\left(x_{1}-x_{0}\right)$ is the theoretical target velocity, and ${\left|\cdot \right|}_{2}^{2}$ is the squared Euclidean norm. This loss function guides the network to learn how to move the mixed state $x_{t}$ along a straight-line direction toward the real data $x_{1}$.

During the inference phase, the model uses the learned velocity field to construct an ODE, calculated as

(9) $$dx_{t}=v_{\theta }\left(x_{t},t,c\right)dt.$$

**Figure 7:**
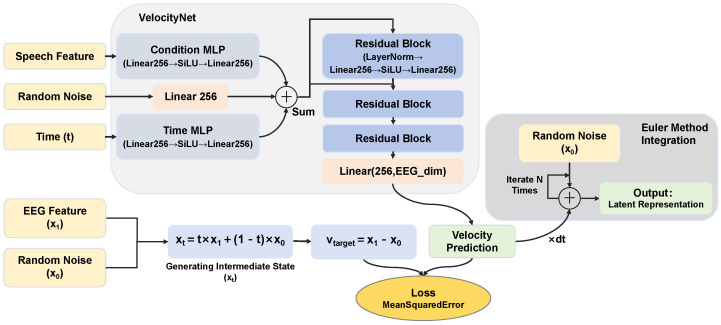
The overall architecture of the Rectified Flow-based generative model.

In order to generate latent representations, the Euler method is used to perform numerical integration of this ODE. Given the conditions *c* and the initial noise $x_{0}$, the state is updated step by step through *N* iterations (set to 10 in this study), calculated as

(10) $$x_{k+1}=x_{k}+\frac{1}{N}v_{\theta }\left(x_{k},\frac{k}{N},c\right).$$

where *k* is the discrete iteration index (0≤k≤N−1); $x_{k}$ is the intermediate flow state at iteration *k* (initialized as $x_{0}$), and $x_{k+1}$ is the updated state after one step; $\frac{1}{N}$ is the step size of Euler’s method; $v_{\theta }\left(x_{k},\frac{k}{N},c\right)$ is the velocity field predicted by the pre-trained network (parameterized by θ), with $\frac{k}{N}$ as the normalized time step and *c* as conditional information. The final state represents the generated latent representations. Thanks to the characteristics of linear flow, Rectified Flow can generate high-quality samples with very few sampling steps and significantly outperform traditional diffusion models. The overall architecture of the Rectified Flow-based generative model is shown in Figure 7.

### 3.4. Fusion and Classification Module

**Figure 8:**
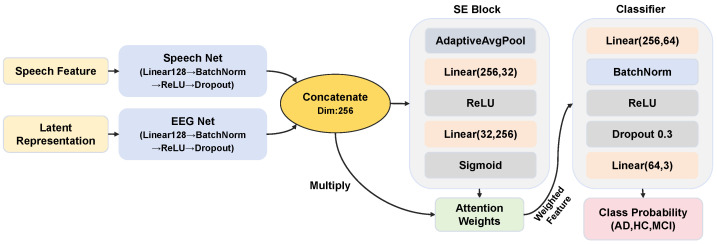
The architecture of the fusion and classification model.

This study constructs a two-stream fusion network incorporating a channel attention mechanism to fully utilize the generated latent representations and real speech features during the classification phase. The model comprises two independent feature encoding branches, which handle speech and generated latent representations respectively. Each branch is composed of fully connected layers, batch normalization, ReLU activation functions, and Dropout layers. The branch maps features of different modalities to a unified semantic space with the dimension set to 128. After encoding, the bimodal features are first concatenated along the channel dimension to form a 256-dimensional joint feature vector. A Squeeze-and-Excitation (SE) module is then introduced to re-weight the feature channels. The specific operations are as follows: first, the features are compressed through global average pooling; then, a bottleneck layer with a compression ratio of 8 is used to learn the interdependencies between channels and generate channel attention weights. By multiplying the weights with the original features element-wise, the model can adaptively enhance the response of key pathological features while suppressing noise-related channels. The classification head adopts a multilayer perceptron (MLP) with 64 neurons in the hidden layer. The weighted fused features are fed into the classification head, which finally outputs the prediction probabilities of each category. The architecture of the fusion and classification model is shown in Figure 8.

## 4. Experiments and Results

To validate the effectiveness of the proposed cross-modal fusion framework, a series of comparative experiments are designed in this chapter. First, the chapter details the evaluation metrics, model parameter configurations, and training strategies, which ensure the reproducibility of the experiments. Secondly, unimodal baseline experiments are conducted to evaluate the respective diagnostic performance of EEG and speech features. Finally, the effectiveness of each module is verified through designed ablation experiments, and the physiological characteristics of the latent space features are analyzed along with a sensitivity analysis of key parameters in our method.

### 4.1. Experimental Setup

This subsection details the various experimental configurations. It outlines the quantitative metrics used for model performance evaluation, alongside the specific implementation details, hyperparameter selections, and training optimization strategies of the deep learning models.

#### 4.1.1. Evaluation Metrics

To comprehensively evaluate the performance of the proposed model on the three-class classification task of AD, HC, and MCI, we adopt accuracy, confusion matrix, sensitivity, specificity, precision, F1 score, and the Area Under the Receiver Operating Characteristic Curve as the core evaluation metrics.

The four statistics in the confusion matrix are defined as follows. True Positives (TP) represent the number of positive samples correctly predicted as the positive class by the model. True Negatives (TN) represent the number of negative samples correctly predicted as the negative class. False Positives (FP) denote the number of negative samples incorrectly predicted as the positive class. False Negatives (FN) denote the number of positive samples incorrectly predicted as the negative class. In the three-class classification task, the aforementioned metrics are calculated using a One-vs-Rest strategy. This approach treats the current category as the positive class and combines all remaining categories into the negative class.

Accuracy is the ratio of all correctly classified samples to the total number of samples. Sensitivity is used to measure a model’s ability to correctly identify positive samples. The mathematical definition of sensitivity is equivalent to that of recall and is calculated as

(11) $$\mathrm{Sensitivity}=\frac{\mathrm{TP}}{\mathrm{TP}+\mathrm{FN}}.$$

Specificity is used to measure a model’s ability to correctly identify negative samples, and it reflects the level at which the model controls the misdiagnosis rate, calculated as

(12) $$\mathrm{Specificity}=\frac{\mathrm{TN}}{\mathrm{TN}+\mathrm{FP}}.$$

Precision is used to measure the proportion of samples predicted as positive by the model that are actually positive, calculated as

(13) $$\mathrm{Precision}=\frac{\mathrm{TP}}{\mathrm{TP}+\mathrm{FP}}.$$

Since there is often a trade-off between precision and recall, the F1 score can comprehensively reflect the overall classification performance of a model on an imbalanced dataset, calculated as

(14) $$F_{1}=2\times \frac{\mathrm{Precision}\times \mathrm{Recall}}{\mathrm{Precision}+\mathrm{Recall}}.$$

The Receiver Operating Characteristic (ROC) curve is plotted with the false positive rate on the horizontal axis and the true positive rate on the vertical axis. This curve demonstrates the dynamic performance of the model under various classification thresholds. The Area Under the Curve (AUC) represents the integral area under the ROC curve. Its value ranges from 0.5 to 1. An AUC value closer to 1 indicates a stronger overall discriminative ability of the model in distinguishing different categories. Furthermore, this metric exhibits strong robustness against sample class imbalance.

In the internal analysis, sensitivity and specificity refer to the individual values calculated for each specific class. For the EEG modality, sensitivity corresponds to the recall rate of the AD category. Specificity corresponds to the recall rate of the healthy controls. Under the speech modality, we calculate the sensitivity and specificity for the AD, MCI, and HC categories separately. The results table explicitly presents the mean and standard deviation of these metrics for each respective category. When comparing our findings with previous research results, the macro-average value is adopted for the recall rate.

#### 4.1.2. Implementation Details and Training Strategy

The training of the Mel-spectrogram feature extraction model adopts the Adam optimizer with an initial learning rate of 0.001 and uses the cross-entropy loss function, with a total of 100 training epochs performed. After the model training is completed, the 128-dimensional vector output by the fully connected layer is extracted as the deep acoustic representation of the Mel spectrogram. The fine-tuning of BERT adopts the AdamW optimizer with a peak learning rate of 2 × 10^−5.^ A mechanism combining linear warmup and linear decay is applied to the learning rate optimization process, with a batch size of 8 and a total of 5 training epochs. Each sample corresponds to a 768-dimensional semantic feature vector.

MAE is independently trained within each fold of cross-validation based on the experimental feature data. The dimensionality reduction target dimension is set according to different features, with a batch size of 64, 100 training epochs, and a learning rate of 0.001. In terms of model architecture, the encoder and decoder introduce Dropout layers to randomly drop neurons, with a Dropout rate of 0.2, and BatchNorm is added after linear layers to stabilize the training distribution. During training, the Adam optimizer with weight decay is used. For each batch of input features, 30% random masking is applied. The encoder encodes the retained features into a low-dimensional vector, and the decoder reconstructs the masked 30% dimensions, with the loss function computed only for the masked areas. An early stopping mechanism is also introduced, monitoring the reconstruction loss on the validation set, and training is stopped if the loss does not decrease for 5 consecutive epochs to avoid overfitting.

In the alignment model, we first independently standardize heterogeneous features to make them have a mean of 0 and a variance of 1, so as to eliminate the dimensional differences between different modalities. In the data loading phase, considering that the EEG and speech data in this study have problems of sample size imbalance and category missing, the experiment designs an asymmetric parallel loading mechanism: speech data is used as the primary modality and loaded in complete batches; EEG data is used as the auxiliary modality and cyclically read through an independent iterator. When speech and EEG samples of the same category coexist in a training batch, the calculation of consistency constraint is triggered. If the EEG data is exhausted, the iterator is automatically reset to ensure the continuous progress of the training process. The alignment network consists of two independent modality encoders and a shared classification and discrimination module. Each encoder is composed of two fully connected layers, where the number of units in the first linear layer is set according to the dimension of input features, and the dimension of the second layer is the dimension of the shared subspace. The first layer of the EEG encoder is set to 512 dimensions for 2335-dimensional EEG features and 64 dimensions for 32-dimensional EEG features. The first layer of the speech encoder uses 256 dimensions, and the shared subspace is fixed at 128 dimensions. Batch normalization and ReLU activation functions are introduced between the encoder layers, with a Dropout rate of 0.2. Finally, both encoders map the input features to a common subspace with the same dimension. In the training configuration, the alignment network adopts the Adam optimizer with an initial learning rate of 0.001 and introduces an L2 weight decay of 0.001 to enhance generalization ability. Every 30 epochs, the learning rate is decayed to 0.5 times its original value. The total number of training epochs is 200. The batch size is 64.

The generative model is trained for 500 epochs with a learning rate of 0.001 and a batch size of 32. Here we employ five-fold cross-validation. The speech dataset is divided into a training set of 280 cases and a test set of 119 cases. Five-fold cross-validation is implemented only on the speech training set. This set is stratified into 5 folds. Each fold takes turns to act as the internal validation set, while the remaining 4 folds serve as the internal training set. The EEG dataset consists of 65 cases. These cases are divided into a training set of 52 cases and a validation set of 13 cases using a fixed 4:1 ratio. This partitioning remains unchanged throughout the entire process of five-fold cross-validation for speech, and it does not rotate with the speech folds. For each speech fold’s internal training set, the fixed EEG training set is paired for model training. The heterogeneous feature alignment network is first trained based on this portion of the data. After the alignment network converges, the speech and EEG aligned features output by the trained alignment network of that fold are used to train the generation model. Upon the completion of model training, the independently trained alignment network and generation model of that fold are used to sequentially perform feature alignment, latent representation generation, and feature fusion classification on the speech fold’s internal validation set. The model parameters for all folds are independently initialized with no parameter sharing between folds. This independent initialization ensures strict separation of training and validation data in each fold. This dual approach from both data partitioning and model training prevents data leakage and ensures the statistical validity of the experimental results.

The hidden layer of the MLP classification model contains 64 neurons, with batch normalization and ReLU activation functions introduced, and the Dropout rate is set to 0.3. The model training process is based on the Adam optimization algorithm and an adaptive learning rate strategy, with a batch size set to 16. Given the small-sample dataset in this study, L2 weight decay regularization and an early stopping mechanism (Patience = 20) based on internal validation set loss monitoring are adopted to suppress overfitting effectively. To eliminate the random bias caused by parameter initialization and data partitioning, the entire validation process is repeated under 5 different random seeds. For each random seed, the mean and standard deviation of each metric across the five folds are calculated as the overall performance of that random seed. Finally, the mean and standard deviation of the results from the 5 random seeds are computed again as the final model evaluation results.

To ensure the scientific rigor and full reproducibility of hyperparameter selection, this study embeds a systematic grid search strategy within a five-fold cross-validation framework. The experiment predefines discrete search spaces for the core hyperparameters. The candidate ranges for the initial learning rates of the alignment network and the generator are set to $\left[1\times 10^{-4},1\times 10^{-2}\right]$ and $\left[5\times 10^{-4},1\times 10^{-3}\right]$, respectively. The candidate set for batch size is {8,16,32,64}. The target dimensions for MAE dimensionality reduction and the shared subspace dimensions of the alignment network are searched within {8,16,32,64,128} and {64,128,256}, respectively. Simultaneously, the network dropout rates are set to {0.2,0.3,0.4,0.5}.

During the specific execution phase of the grid search, each fold of cross-validation strictly reserves 10% of the current training set as an internal validation set. The model is fitted on the remaining 90% of the training set with various parameter combinations. It is then evaluated on the internal validation set. This evaluation relies on a combined criterion of classification accuracy, macro-averaged F1 score, and early-stopping monitored loss to select the best-performing parameter combination for that fold. After the completion of five rounds of within-fold search, the frequencies of winning hyperparameter combinations are tallied. The combination with the highest frequency is established as the final global experimental parameter set. Once locked in by the grid search, the global parameters remain strictly fixed in subsequent repeated experiments based on five different random seeds. They also remain fixed during the final evaluation on an independent test set. This scheme completely prevents potential data leakage from the hyperparameter tuning process to the test set. Consequently, it provides a reproducible standardized procedure for future research.

### 4.2. Performance Analysis of Single-Modality Baselines

In order to highlight the advantages of cross-modal integration, it is first necessary to establish the performance benchmarks for single modalities. This subsection independently evaluates the diagnostic performance of EEG features and speech features, and analyzes the contribution and limitations of the two types of biological signals in AD diagnosis.

#### 4.2.1. EEG Feature Performance

We first explored the effectiveness of single-domain EEG features. The binary classification performance of AD and healthy controls is shown in Table 3.

Among the traditional handcrafted features reduced via PCA, MSE exhibited relatively superior discriminative power and achieved an accuracy of 0.77 and a high sensitivity of 0.89. This indicates that nonlinear complexity features of EEG signals are highly sensitive to the abnormal neural activity in AD patients. However, the specificity of this feature was relatively low with a large standard deviation, which suggested a high misclassification rate for the HC group and poor model stability across different data partitions. In contrast, the PLI demonstrated a well-balanced performance, with all three metrics exceeding 0.7 and relatively small standard deviations. This confirms that abnormal brain network connectivity serves as a robust biological marker for AD diagnosis. The PSD topographic features extracted by MobileNetV2 achieved the optimal performance among all single-modality features, with an accuracy of 0.82, sensitivity of 0.81 and specificity 0.84. This suggests that through transfer learning and fine-tuning strategies, the model effectively overcame the overfitting and random fluctuations associated with small-sample datasets, extracting deep semantic features with high generalization capability.

To verify the complementarity between EEG features from different domains and to explore the optimal feature fusion scheme, we compared two strategies: hierarchical dimensionality reduction fusion (where each domain’s features are reduced independently before concatenation) and joint feature fusion (where raw features from all domains are concatenated before reduction). The performance of PCA and MAE in compressing these high-dimensional joint features was also analyzed, with results presented in Table 4. The input features include time-domain, frequency-domain, time–frequency domain, MSE, PLI, and PSD topographic features. The total dimension of the original joint features is 2335. The multidimensional EEG feature set is abbreviated as TFDMPI.

Experimental results showed that hierarchical dimensionality reduction fusion achieved an accuracy of 0.70. While this method avoids the direct reduction in high-dimensional joint features, its performance is relatively poor. This may be because hierarchical reduction disrupts the latent nonlinear correlations between features from different domains, leading to the early loss of complementary information. Within the joint feature fusion strategy, applying PCA caused the accuracy to drop to 0.62 and specificity to only 0.55. This indicates that the joint distribution of multi-domain features possesses a highly nonlinear structure and simple linear orthogonal projection cannot effectively decouple complex dependencies, which results in the loss of critical discriminative information.

In contrast, using MAE to compress high-dimensional joint features significantly enhanced model performance. When the latent dimension was set to 32, the accuracy reached 0.86 with a specificity of 0.90 and relatively low standard deviations. Compressing features to 32 dimensions outperformed the 64-dimension configuration, a phenomenon consistent with Occam’s Razor and the principles of small-sample learning. Given the limited sample size, a lower-dimensional feature space implies fewer model parameters and a reduced risk of overfitting. The 32-dimensional representation not only removes redundant noise but also forces the model to focus on the most distinctive core pathological patterns, which achieves optimal generalization performance.

#### 4.2.2. Speech Feature Performance

The classification performance of acoustic features is presented in Table 5.

Directly utilizing the high-dimensional raw ComParE feature set led to the “curse of dimensionality,” and resulted in low overall accuracy and a significant tendency for missed diagnoses. After introducing an MAE for feature reconstruction, the accuracy improved to 0.80, and the sensitivity for AD increased to 0.70. This suggests that the core pathological information in speech signals used to distinguish cognitive impairment is actually distributed within a lower-dimensional manifold space. The raw HuBERT features exhibited the best classification performance, with an accuracy of 0.85 and both sensitivity and specificity exceeding 0.84. This demonstrates that large-scale pre-trained self-supervised models can effectively capture pathological information. Furthermore, the high-dimensional feature space did not trigger severe overfitting, as seen with traditional handcrafted features, and reflected the high robustness of pre-trained representations. When reduced to 32 dimensions via MAE, the performance remained high at 0.84, close to the raw feature level, which facilitates dimension control during subsequent multimodal fusion. Deep features from Mel-spectrograms (Melspec) achieved an accuracy of 0.81, with AD sensitivity reaching 0.86 and HC specificity reaching 0.98. This indicates that the time–frequency image features extracted by the 2DCNN are highly stable and possess significant pathological characteristics.

The classification performance of textual features is summarized in Table 6.

Semantic classification based on FunASR automatic transcriptions achieved an accuracy of 0.74, outperforming the manual text baseline. This improvement can be attributed to the FunASR pipeline’s ability to preserve more pathological markers during audio processing, such as hesitations and fragmented speech, which are often corrected during manual transcription. These elements, rather than being noise, provide auxiliary diagnostic information at the semantic level. This result strongly supports the clinical potential of our fully automated speech recognition pipeline. Furthermore, when the 768-dimensional fine-tuned BERT features were compressed to 64 dimensions by MAE, the model accuracy remained at 0.74, consistent with the performance of the full-dimensional features. Similarly, reducing TF-IDF statistical features to 32 dimensions via PCA maintained the same performance level as the original features, with an accuracy of 0.66.

This study constructed a 294-dimensional multimodal speech feature set (CHMLTB) comprising ComParE (32-dim), HuBERT representation (32-dim), deep Mel-spectrogram features (128-dim), lexical features (6-dim), TF-IDF (32-dim), and semantic features (64-dim). We investigated the effectiveness of two fusion strategies, direct concatenation and feature reorganization based on MAE, with the classification metrics presented in Table 7.

Experimental results indicate that the direct concatenation strategy achieved an accuracy of 82.02%. Although this performance surpasses the single-modality textual and Mel-spectrogram baselines, it remains lower than the peak performance of the single-modality HuBERT features. This suggests that simple feature stacking fails to fully exploit the complementary advantages between modalities. In the absence of feature space alignment, the weaker textual modality can trigger a negative transfer effect and interfere with the decision boundaries of strong acoustic features. After introducing MAE for feature reorganization, the model’s accuracy improved to 82.86%. While this limited increase suggests that the current combination of speech and text features is approaching a performance saturation point, it is noteworthy that the standard deviation across all evaluation metrics significantly decreased. Although the fusion strategy faces a bottleneck in improving absolute accuracy, it effectively suppresses the random fluctuations inherent in single modalities, which achieves more balanced and reliable classification performance.

### 4.3. Speech—Latent Representation Fusion Performance Analysis

This section evaluates the effectiveness of the proposed generative cross-modal fusion strategy. First of all, the experiments quantitatively assess the performance of feature alignment. Next, t-SNE visualizations illustrate the distribution of the feature space before and after alignment. Finally, we integrate acoustic features with latent representations to demonstrate the final performance of AD detection.

#### 4.3.1. Feature Alignment Evaluation

To investigate the effects of different input feature dimensions and common subspace capacities on heterogeneous feature alignment, this study designed five sets of comparative experiments. These experiments examined the combined performance of EEG features (TFDMPI) and speech features (CHMLTB) in both their original and reduced dimensions and mapped the input features into latent spaces of varying capacities for testing. The alignment performance under different feature dimensions and subspace capacities is presented in Table 8.

The results show that after compressing the EEG features from 2335 dimensions to 32, the model accuracy experienced only marginal fluctuations. This confirms that low-dimensional EEG representations provide a sufficiently robust supervisory signal while effectively eliminating redundant information from the raw data. In contrast, the 128-dimensional speech features optimized via MAE performed significantly better than the high-dimensional raw features, which suggests that reducing input heterogeneity facilitates the rapid convergence of the alignment network. Regarding the subspace capacity, the model performance followed a trend of initially increasing and then decreasing. A sparse distribution in a 256-dimensional space weakened the effectiveness of metric constraints, whereas excessive compression in a 64-dimensional space led to the loss of semantic information. The model achieved its optimal performance with an accuracy of 0.8689 when the subspace was set to 128 dimensions. This configuration realizes an isometric mapping between the input and latent spaces, maximizing the preservation of the feature manifold structure. Ultimately, the speech features enhanced through alignment achieved a sensitivity of 0.9029 in the AD group and a specificity of 0.9892 in the HC group. These results demonstrate superior performance that surpasses the baselines of single-modality speech features.

Further analysis of the confusion matrix under the optimal configuration revealed that misclassified samples were concentrated primarily between the AD and MCI categories. Specifically, an average of 6.6 MCI cases were misidentified as AD, while 3.2 AD cases were classified as MCI. This confusion aligns with the progression characteristics of the Alzheimer’s Disease Continuum and reflects the objective pathological overlap in neurophysiological and acoustic representations between MCI, as a prodromal stage of dementia, and confirmed AD. In contrast, the average number of cases where the pathological groups (AD and MCI) were underdiagnosed as HC was minimal, at only 0.2 and 0.6 cases, respectively. This exceptionally low false-negative rate suggests that the alignment model effectively captures abnormal neurodegenerative signals and distinguishes them from healthy physiological patterns. These results demonstrate that cross-modal feature alignment guided by unpaired EEG data compensates for the limitations of the speech modality in deep pathological semantic representation, which significantly enhances overall diagnostic performance.

#### 4.3.2. Visualization of Feature Spaces

**Figure 9:**
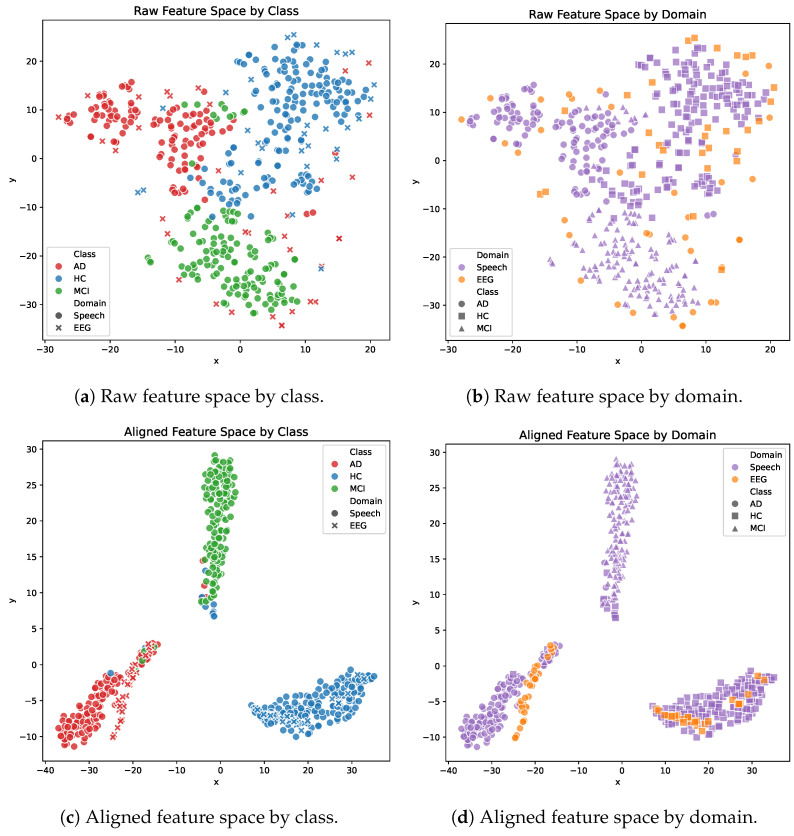
Feature space distribution visualization.

In order to intuitively assess the effectiveness of the proposed adversarial metric-constrained network in eliminating modality differences and extracting discriminative features, we used the t-SNE algorithm to perform visualization on the original input features and the aligned latent space features, as shown in Figure 9.

Regarding the category distribution (Figure 9a vs. Figure 9c), the constraints imposed by the center loss have significantly enhanced intra-class compactness. Samples that were originally loose and overlapping have been reorganized into independent clusters with clear boundaries, effectively reducing the difficulty of classification decisions. Of particular importance is the behavior of the MCI samples; despite the lack of EEG supervision, they adaptively clustered within the manifold transition region between AD and HC. This effectively validates the adaptive interpolation strategy proposed in this study. Comparing the feature distributions before and after alignment (Figure 9b vs. Figure 9d), it is evident that adversarial training has led to a deep integration of the previously fragmented speech and EEG distributions. The modality boundaries have completely vanished, and this proves that the model has successfully stripped away the physical attributes unique to each modality and extracted cross-modality shared semantic representations. In summary, the alignment framework constructed in this research successfully achieves a representation migration from physical-feature-dominated to pathological-semantic-dominated, which establishes a common subspace that is both modality-invariant and highly discriminative.

#### 4.3.3. Ablation Study

To systematically evaluate the effectiveness of each key module, we conducted an ablation comparison on the alignment model, the generative model, and fusion strategies. Table 9 presents the experimental results. The remaining relevant indicators are listed in detail in Table A1 of Appendix A. The results demonstrate that the simultaneous introduction of feature alignment and generation mechanisms maximizes the classification efficacy of the model.

For the validation of the generative module, the experiment compared the baseline aligned features (Align-CHMLTB), the aligned features with random noise (Align-CHMLTB + Random-Noise), and the aligned features combined with the generated latent representation (Align-CHMLTB + Latent Representation). The data revealed that the model accuracy was 0.8689 under the sole use of the alignment model. After directly injecting random noise into the aligned features, the model’s performance significantly deteriorated, with accuracy dropping to 0.7782, cross-fold variance sharply increasing to 0.0909, and the sensitivity of the MCI group falling to 0.5128, which indicate that meaningless random perturbations severely disrupted the original semantic boundaries. By contrast, after the introduction and direct concatenation of the latent representation from the generative model, the accuracy improved to 0.8773, and the F1 score reached 0.8726. This comparison directly rules out the possibility that the performance gain originates from an increase in feature dimensions or noise regularization effects. It verifies that the latent representation, which is based on feature alignment and Rectified Flow generation, truly encodes pathological semantic information with high clinical discriminability.

The analysis of the synergistic effect between the alignment and generative modules found that unaligned raw speech features directly passed through the generative model (CHMLTB-128 + Latent Representation) achieved an accuracy of 0.8672. This value was slightly lower than the alignment baseline, and it was also lower than the joint architecture of alignment followed by generation (0.8773). The finding demonstrates that cross-modal latent space alignment is a necessary prerequisite for the generative network to effectively reconstruct pathological features. Furthermore, reliance solely on the generated latent representation for classification (Latent Representation) yielded an accuracy of only 0.7210. Therefore, the generated physiological representation cannot completely replace the original acoustic features, and the relationship between the two is complementary rather than substitutional.

In the exploration of feature fusion strategies, the introduction of the attention mechanism (Align + Flow + Attention) further optimized the model performance compared to direct concatenation (accuracy of 0.8773). The accuracy and F1 score of the attention fusion architecture reached the optimal values of 0.8908 and 0.8862, respectively. Additionally, the distribution of sensitivity and specificity across the three categories became more balanced. This result shows that the attention mechanism adaptively calibrates the weights between the aligned speech features and the generated physiological representations. It effectively suppresses redundant information and achieves the optimal fusions.

**Figure 10:**
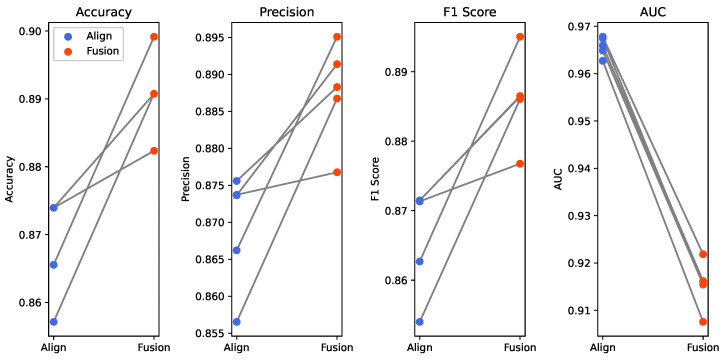
Comparison chart of key metrics for the alignment and fused model with five different seeds. In the chart, blue dots represent the performance metrics of the alignment model under a single seed, and red dots represent the performance metrics of the fused model.

We further employed the Wilcoxon signed-rank test to assess the statistical significance of performance differences between the single alignment model (Align) and the fused alignment–flow model (Align + Flow + Attention). As a nonparametric method, it is suitable for comparing paired samples without assuming normality. The test ranks the differences between paired observations and uses their signs to evaluate statistical significance, reported via the test statistic and *p*-value (*p* < 0.05 indicates significance). We performed pairwise Wilcoxon tests on accuracy, precision, F1 score, and AUC across five random seeds, with metric comparisons visualized in Figure 10. The fused model achieved statistically superior performance in accuracy, precision, and F1 score (all Wilcoxon statistic = 15.0000, *p* = 0.03125). In contrast, no significant difference was observed for AUC (statistic = 0.0000, *p* = 1). Although incorporating the generated physiological latent representations improved threshold-dependent accuracy and F1 score by complementing missing pathological information, fusion with speech features may have increased overlap in the global predictive distributions, which led to a slight, statistically insignificant decrease in AUC. Overall, the performance improvements of the fused model are statistically significant and further validate the effectiveness of the generated latent space representation.

#### 4.3.4. Spectrum Analysis of Latent Representation

This section systematically compared the spectral similarities and differences between real EEG signals and generated latent representations. We employed statistical testing (*t*-tests) to mark significant differences between the two sets of signals at each frequency point. This evaluation determines whether the generated representations successfully retained the neurophysiological features of the real EEG.

**Figure 11:**
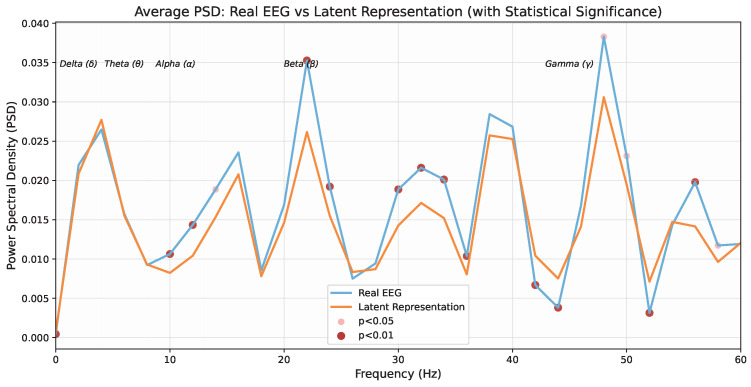
Comparison and statistical significance labeling of average PSD between real EEG and latent representations.

Figure 11 shows that the real EEG (blue curve) and the latent representation (orange curve) exhibit similar trends across most frequency ranges, which means the generated features successfully fit the energy distribution of the real EEG in terms of the overall spectral structure. Red dots (*p* < 0.05) and dark red dots (*p* < 0.01) indicate statistically significant or highly significant differences in PSD between the two groups of signals at these frequency bins, respectively. The limited number and scattered distribution of these significant points show that no significant differences exist in most frequency bands. The latent representation exhibits high consistency with real EEG in spectral characteristics and captures the neurophysiological properties of AD patients.

#### 4.3.5. Parameter Analysis

This subsection establishes the optimal hyperparameter configuration for the loss weights of the alignment network and the inference steps of the flow model through a systematic parameter sensitivity analysis. Concurrently, we combine the analysis of the total model parameter count and effective capacity. This analysis further demonstrates the structural rationality and generalization reliability of the proposed framework under the current scale of small medical datasets.

**Figure 12:**
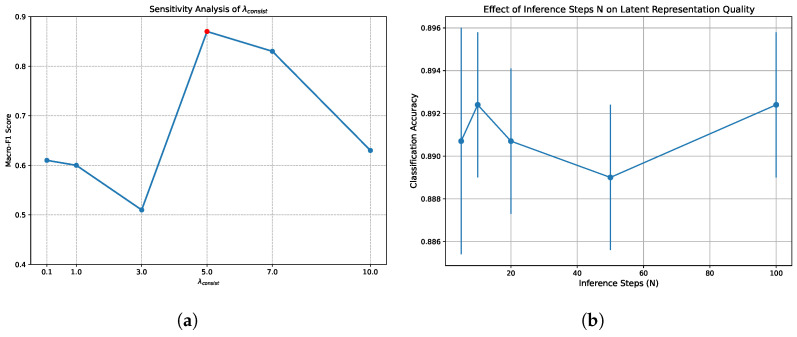
Parameter analysis visualization. (**a**) Sensitivity analysis of $\lambda_{\mathrm{consist}}$. The horizontal axis represents the $\lambda_{\mathrm{consist}}$ value, and the vertical axis represents the macro-average F1 score of the internal validation set. (**b**) The impact of different inference steps *N* on the classification performance of the fused speech and latent representation features. The blue line and error bars represent the average classification accuracy and standard deviation under different *N* values.

We conducted a systematic sensitivity analysis and grid search on the loss weights in the alignment strategy based on an internal validation set. Take the consistency constraint weight $\lambda_{\mathrm{consist}}$, which determines the strength of multimodal alignment, as an example. Under the premise of controlling other variables as fixed, the experiment set its search interval to {0.1,1.0,3.0,5.0,7.0,10.0}. Figure 12a illustrates the changes in the macro-average F1 score of the model on the internal validation set according to the variations in $\lambda_{\mathrm{consist}}$. When $\lambda_{\mathrm{consist}}$ was less than 3.0, the bimodal features failed to achieve effective geometric alignment in the latent space due to the weak gradient signal of the consistency constraint and caused a lower F1 score for the model. When the weight increased to 5.0, the F1 score jumped to a peak of approximately 0.87, which proves that the network achieved the optimal solution between enforcing modal alignment and ensuring classification discriminability at this specific point. However, when $\lambda_{\mathrm{consist}}$ further increased to 10.0, the excessively strong consistency penalty triggered over-regularization. It interfered with the decision boundary learning of the main classifier and forced the performance to drop back to around 0.63. This systematic validation established the optimality of setting $\lambda_{\mathrm{consist}}$ to 5.0 at the experimental level. Similarly, the domain-adversarial loss weight $\lambda_{\mathrm{domain}}$ and the center loss weight $\lambda_{\mathrm{center}}$ followed the exact same systematic search strategy. The experimental data confirmed that a $\lambda_{\mathrm{domain}}$ of 0.1 successfully maximized the confusion of modal sources without losing feature semantics. Meanwhile, a $\lambda_{\mathrm{center}}$ of 0.05 effectively prevented the excessive collapse of the feature space and provided moderate intra-class convergence. Once the inner-fold validation set search locked all weight parameters, they remained strictly fixed in the subsequent performance evaluations on the test set.

We systematically investigated the impact of different inference steps *N* (5, 10, 20, 50, 100) on the quality of the generated features. Figure 12b shows that as *N* increased, the overall classification accuracy remained stable with a small standard deviation. The accuracy reached its peak when *N* was 10, and the fluctuation was minimal. A further increase in the inference steps did not bring significant performance improvements; instead, it increased the computational overhead. Therefore, the selection of 10 as the inference step count ensures generation quality, numerical stability, and inference efficiency. This experiment fully verifies that the Rectified Flow model can achieve highly efficient and stable latent representation generation under a low number of steps.

Based on the characteristics of small-sample medical datasets, this study has strictly controlled the capacity of the model architecture. The total number of learnable parameters in the proposed framework is approximately 0.79 M (specifically: alignment module 159,364; generation module 577,056; fusion module 54,403). Although the absolute number of parameters numerically exceeds the size of the training samples, the effective capacity of the model is strictly constrained through network design and training strategies, preventing the risk of overfitting in small-sample scenarios.

Our architecture abandons the end-to-end direct signal connection mode. The 0.79 M updatable parameters are only deployed in a highly condensed deep semantic space, and significant structural information bottlenecks are introduced within the network. For example, the cross-modal shared subspace is strictly compressed to 128 dimensions, and EEG features are purified into a compact 32-dimensional manifold using MAE. This low-dimensional mapping forces the model to filter redundant information, resulting in an actual degree of freedom much lower than the absolute number of parameters. At the optimization level, the training process incorporates multiple explicit regularization methods, including L2 weight decay, Dropout neuron random deactivation, and early stopping strategies based on an independent validation set. More importantly, the ordinary differential equation solving process in the Rectified Flow framework requires the model to fit a continuous, smooth probability flow trajectory. This dynamic property imposes a strong geometric smoothness prior in the latent space, effectively suppressing the common decision boundary fragmentation problem caused by over-parameterization. The above effective capacity constraint mechanisms together ensure the model’s robust generalization capability under five-fold cross-validation.

## 5. Discussion

This section conducts an in-depth discussion across three progressive levels. First, it compares the core findings of this study with the latest achievements in existing literature and elucidates the methodological innovations and performance advantages of our research. Second, it systematically analyzes two types of uncertainties present in the research process and explores their potential impact on the experimental results. Finally, it summarizes the limitations of this study and proposes specific recommendations for future research directions based on the current findings.

### 5.1. Results Discussion and Comparison

This study first validated the benchmark status of single-modal, multi-dimensional EEG features in AD detection. Results indicate that the EEG modality, with its ability to directly map neuropathology, exhibits extremely high accuracy and specificity in the binary classification task of distinguishing AD from HC. Specifically, MSE features effectively captured the phenomenon of EEG rhythm slowing and complexity loss caused by the depletion of cholinergic neurons in patients. Meanwhile, functional connectivity features constructed based on the PLI revealed impairments in fronto-temporal connectivity. However, despite the high pathological correlation of EEG features, the requirements for professional equipment and operators, combined with the potential discomfort caused to patients during acquisition, objectively limit its potential for large-scale community screening scenarios. Consequently, achieving high-precision computer-aided diagnosis using convenient modalities has become an imperative task.

In contrast, single-modal speech features offer the advantage of low-cost acquisition but suffer from significant performance bottlenecks. As a behavioral proxy for cognitive function, speech can reflect a subject’s cognitive load and linguistic impairment through texture details in Mel-spectrograms or vocabulary poverty. However, speech signals are inherently susceptible to interference from non-pathological factors such as environmental noise, dialect differences, and education levels, which result in high data heterogeneity. Particularly for MCI patients, relying solely on speech features makes it difficult to capture early cognitive decline, and the sensitivity in the MCI group is lower than that of other categories. Relying on a single speech modality is insufficient to establish a robust pathological mapping; thus, there is an urgent need to introduce complementary information to enhance its discriminative power.

To address these limitations, the cross-modal generation and fusion framework proposed in this study successfully breaks the performance boundaries of single-modal speech. By constructing a heterogeneous feature alignment network, the model first strips away modality-specific physical attributes in the latent space and employs adversarial learning to extract cross-modal shared pathological semantic representations. To address the challenge of missing paired MCI EEG data, the proposed adaptive interpolation strategy plays a decisive role. Relying on the manifold continuity hypothesis of deep networks, the model utilizes AD and HC samples as semantic anchors to successfully map unsupervised MCI speech samples to the transition region between them, and reconstructs the complete pathological evolution trajectory in the latent space. This mechanism enables the model to infer the precise position of a sample within the pathological manifold during inference based on faint cues in the speech, and effectively corrects the blurred boundaries often encountered in traditional MCI detection methods.

The latent representations generated based on Rectified Flow play a crucial role in physiological information completion during the fusion stage. These generated latent representations are not random noise but contain deterministic neurophysiological priors derived from speech features. By integrating the behavioral representations of speech with the speculative physiological evidence from latent representations, the fusion model achieves a complementary enhancement of multi-view information. Experimental data confirm that this fusion strategy not only surpasses the best performance of single-modal EEG in accuracy but also significantly reduces the standard deviation across various metrics, demonstrating the system’s robustness when facing complex samples. This result holds significant clinical implications, as it implies that our method retains the portability of speech screening while largely replicating the high reliability of EEG detection. The framework proposed in our study provides a viable technical path for low-cost, high-precision early screening of Alzheimer’s disease.

Table 10 presents a performance comparison between the proposed method and existing advanced techniques using the same speech dataset.

Experimental results demonstrate that the fusion framework proposed in our study achieved significant superiority across all evaluation metrics. Compared to the official baseline system, our method improved accuracy by 9.28%, substantially outperforming traditional models that rely solely on shallow acoustic features. By leveraging the complementary physiological information provided by the generated latent representations, our method effectively overcomes the performance bottlenecks inherent in existing single-modality or simple fusion approaches, and achieves high-precision AD detection.

### 5.2. Uncertainty Analysis

The uncertainties in this study primarily originate from two aspects: input data and model parameters. Both aspects can potentially affect the stability and generalization of the experimental results.

#### 5.2.1. Uncertainty of Input Data

The EEG data used in this study belong to a public dataset that underwent standardized preprocessing by researchers such as Miltiadous et al. [20]. During the acquisition phase, the raw data effectively controlled blink artifact interference through an eyes-closed resting state. The original authors also completed preprocessing operations such as 0.5–45 Hz Butterworth bandpass filtering, Artifact Subspace Reconstruction, and Independent Component Analysis. These steps significantly eliminated explicit artifacts and noise, such as muscle movements, power line interference, and baseline drift. However, EEG signals are physiological electrical signals with high spatiotemporal resolution. Implicit interferences, such as slight changes in electrode contact and weak physiological noise during the acquisition process, are difficult to eradicate completely [45]. Such residual signal disturbances become potential sources of uncertainty in EEG data and may have a slight impact on the accuracy of EEG feature extraction. Simultaneously, the EEG data used in this study are short-term records under a resting state. Compared with long-term EEG monitoring, the captured EEG features have sample limitations. They cannot comprehensively reflect the dynamic change characteristics of the subjects’ EEG activities. This limitation further increases the uncertainty at the data level. In addition, individual factors such as the subjects’ pronunciation states and emotional fluctuations can also lead to subtle differences in speech features. These factors serve as another source of input data uncertainty.

#### 5.2.2. Uncertainty of Model Parameters

This framework involves the setting of multiple key model parameters within core modules like feature alignment, generation, and fusion. Examples include the dimension selection for feature dimensionality reduction, the learning rate of the generative model, and the weight allocation of the fusion strategy. The experiments verified and determined the optimal values for all these parameters multiple times. Although this process ensured the optimality of the experimental results, a certain degree of parameter tuning uncertainty still exists. Different parameter combinations may cause subtle fluctuations in model performance. Furthermore, this study has not yet conducted a comprehensive sensitivity analysis for all core parameters. It cannot accurately quantify the impact degree of a single parameter change on the overall model performance.

### 5.3. Research Limitations and Prospects

Although the Rectified Flow-based cross-modal feature generation framework proposed in this study has achieved significant performance improvement in early screening of AD, and the generated latent representations objectively conform to typical EEG pathological patterns at the group level, there are still the following limitations that need to be addressed in future work due to objective constraints.

The speech and EEG data in this study originate from independent distributions. The research lacks a speech–EEG paired dataset synchronously collected from the same subjects. Therefore, the cross-modal latent representation generated by the Rectified Flow model is essentially a physiological dimension completion of the speech modality’s pathological semantics. It is not a replication of the individual’s actual physical EEG signals. Because the study lacks a control gold standard at the individual level, it is currently difficult to absolutely quantify the mapping error between the generated features and the real physiological signals on a microscopic scale.

As shown in Table 11, the two datasets used in this study exhibit significant differences in ethnicity, language, and cognitive assessment protocols. There are inherent cross-domain transfer limitations when using neurophysiological priors from European populations to guide speech AD detection in the Chinese population. First, language features, especially prosody and word choice, are greatly influenced by cultural background and education level. Although the neurophysiological degeneration caused by AD is biologically universal, the clinical manifestations of expressive language impairment may vary slightly between different languages, which increases the difficulty for the alignment network to remove population-specific physical features. Second, physical differences, such as skull thickness and head proportions, may affect the spatial distribution of EEG signals. Therefore, topological features learned from non-Chinese datasets may show spatial ‘misalignment’ when applied to Chinese populations, potentially leading to slight inaccuracies in the topology of the generated latent space representations. Finally, the two datasets use different assessment tools and inclusion criteria (such as differences in MMSE score thresholds). This asymmetry means that the ‘AD semantics’ learned by the model are not necessarily strictly equivalent between datasets, which may affect the fitting accuracy of the conditional velocity field of pathological trajectories.

The effective clinical sample size of the current study is relatively small. Furthermore, the data collection environment remains highly ideal. The robustness of the model under broader dialect distributions, complex clinical background noise environments, and multi-disease comorbidity conditions still requires thorough validation.

In response to these limitations, future research will focus on collaborative studies with clinical institutions. Our future work will construct a high-quality, synchronous speech–EEG paired dataset and will also further optimize the fine-grained cross-modal alignment capability of the flow matching network. These steps will accomplish the critical transition from “pathological semantic completion” to “individual precise physiological mapping.” Additionally, subsequent studies will evaluate the generalization efficacy of the model across larger-scale, cross-lingual, and strong-noise datasets. The research will also explore the introduction of other non-invasive behavioral modalities, such as facial expressions and eye movements. This expansion will build a more comprehensive and objective multi-view cognitive degradation assessment system.

## 6. Conclusions

To address the clinical pain points of limited accuracy in single-modal speech detection and the scarcity of paired multimodal data in early AD screening, this paper proposes a latent representation generation and enhanced detection framework based on feature alignment and Rectified Flow. The main contributions of this study are reflected in the following three aspects. First, this paper constructs a heterogeneous feature alignment network, which successfully resolves the challenge of cross-modal data distribution heterogeneity. By introducing metric constraints and domain adversarial mechanisms, the model maps speech and EEG features—which differ vastly in physical attributes—into a common semantic subspace that possesses modality invariance. Furthermore, this study utilizes a conditional Rectified Flow model to generate latent representations, which are rich in neurophysiological priors. Finally, a channel attention mechanism is employed to fuse the aligned speech features with the latent representations and achieves a detection accuracy of 89.08%. The sensitivity for the AD group reaches 88%, and the specificity reaches approximately 92%. These results significantly outperform the efficacy of single-modal speech features and demonstrate the effectiveness of the proposed method, which provides a new perspective for low-cost early AD detection.

## Figures and Tables

**Table 1:** Duration of individual samples and number of each category.

Type	Duration	AD	MCI	HC	Total
Training set	28–60 s	79	93	108	280
Test set	44–60 s	35	39	45	119

**Table 2:** Dimensionality changes of EEG and speech features.

Feature	Method	Primitive Dim	Low Dim
Time-domain	PCA	133	13
Frequency-domain	PCA	190	8
Time–frequency	PCA	342	37
MSE	PCA	380	8
PSD maps	MAE	1280	16
ComParE	MAE	6373	32
HuBERT	MAE	1024	32
TF-IDF	PCA	1000	32
Semantic (BERT)	MAE	768	64

**Table 3:** Performance analysis of single-modality EEG features.

Feature	Model	Accuracy	Sensitivity	Specificity	AUC
Time-domain	PCA	0.69±0.07	0.95±0.07	0.39±0.15	0.67±0.18
Frequency-domain	PCA	0.74±0.12	0.83±0.17	0.61±0.30	0.82±0.16
Time–frequency	PCA	0.63±0.08	0.61±0.25	0.65±0.15	0.63±0.09
Multiscale Entropy	PCA	0.77±0.13	0.89±0.17	0.61±0.30	0.92±0.12
Phase Lag Index	/	0.74±0.08	0.75±0.19	0.72±0.14	0.73±0.08
PSD Image	MobileNetV2 + MAE	0.82±0.03	0.81±0.04	0.84±0.08	0.90±0.04

**Table 4:** Comparison of different feature fusion strategies and dimensionality reduction methods on EEG features.

Feature	Fusion Strategy	Model	Dim	Accuracy	Sensitivity	Specificity	AUC
TFDMPI-92	Hierarchical Fusion	/	92	0.70±0.08	0.72±0.07	0.67±0.18	0.76±0.09
TFDMPI-22	Joint Fusion	PCA	22	0.62±0.09	0.67±0.19	0.55±0.06	0.63±0.07
TFDMPI-64	Joint Fusion	MAE	64	0.83±0.06	0.80±0.08	0.86±0.07	0.90±0.04
**TFDMPI-32**	**Joint Fusion**	**MAE**	**32**	0.86±0.04	0.84±0.05	0.90±0.06	0.93±0.02

**Table 5:** Classification performance of acoustic features (metrics for AD/HC/MCI are stacked vertically).

Feature	Method	Dim	Accuracy	Sensitivity (AD/HC/MCI)	Specificity (AD/HC/MCI)	Confusion Matrix
ComParE	/	6373	0.72±0.04	0.44±0.05	0.88±0.03	$\left[\begin{array}{ccc} 15.4 & 10.0 & 9.6\\ 3.6 & 39.6 & 1.8\\ 6.6 & 1.6 & 30.8 \end{array}\right]$
				0.88±0.07	0.84±0.04	
				0.79±0.02	0.86±0.02	
ComParE	MAE	32	0.80±0.01	0.70±0.05	0.88±0.01	$\left[\begin{array}{ccc} 24.6 & 4.0 & 6.4\\ 3.8 & 39.6 & 1.6\\ 6.6 & 1.2 & 31.2 \end{array}\right]$
				0.88±0.02	0.93±0.02	
				0.80±0.02	0.90±0.01	
**HuBERT**	**/**	**1024**	0.85±0.02	0.89±0.05	0.88±0.02	$\left[\begin{array}{ccc} 31.2 & 2.6 & 1.2\\ 4.4 & 37.8 & 2.8\\ 5.8 & 0.6 & 32.6 \end{array}\right]$
				0.84±0.02	0.96±0.03	
				0.84±0.03	0.95±0.03	
HuBERT	MAE	32	0.84±0.01	0.81±0.04	0.89±0.01	$\left[\begin{array}{ccc} 28.4 & 1.6 & 5.0\\ 4.2 & 38.6 & 2.2\\ 5.0 & 0.8 & 33.2 \end{array}\right]$
				0.86±0.01	0.97±0.01	
				0.85±0.03	0.91±0.01	
Melspec	2DCNN	128	0.81±0.00	0.86±0.01	0.84±0.00	$\left[\begin{array}{ccc} 30.2 & 0.6 & 4.2\\ 6.0 & 35.2 & 3.8\\ 7.2 & 1.0 & 30.8 \end{array}\right]$
				0.78±0.01	0.98±0.01	
				0.79±0.01	0.90±0.01	

**Table 6:** Classification performance of textual features (metrics for AD/HC/MCI are stacked vertically).

Input	Model	Dim	Accuracy	Sensitivity (AD/HC/MCI)	Specificity (AD/HC/MCI)	Confusion Matrix
Manual Text	BERT	768	0.69±0.01	0.53±0.04	0.89±0.02	$\left[\begin{array}{ccc} 18.6 & 13.8 & 2.6\\ 5.6 & 37.6 & 1.8\\ 3.4 & 10.0 & 25.6 \end{array}\right]$
				0.84±0.01	0.68±0.03	
				0.66±0.01	0.94±0.01	
FunASR Text	BERT	768	0.74±0.01	0.60±0.04	0.93±0.01	$\left[\begin{array}{ccc} 21.0 & 5.8 & 8.2\\ 3.6 & 37.6 & 3.8\\ 2.0 & 7.6 & 29.4 \end{array}\right]$
				0.84±0.01	0.82±0.01	
				0.75±0.01	0.85±0.03	
**FunASR Text**	**BERT + MAE**	**64**	0.74±0.01	0.54±0.04	0.96±0.01	$\left[\begin{array}{ccc} 18.8 & 7.0 & 9.2\\ 0.8 & 39.4 & 4.8\\ 2.6 & 6.8 & 29.6 \end{array}\right]$
				0.88±0.02	0.81±0.03	
				0.76±0.01	0.82±0.01	
TF-IDF	/	1000	0.66±0.02	0.38±0.05	0.92±0.03	$\left[\begin{array}{ccc} 13.4 & 14.6 & 7.0\\ 5.0 & 35.8 & 4.2\\ 2.0 & 8.0 & 29.0 \end{array}\right]$
				0.80±0.03	0.69±0.05	
				0.74±0.04	0.86±0.03	
TF-IDF	PCA	32	0.66±0.02	0.38±0.05	0.92±0.03	$\left[\begin{array}{ccc} 13.4 & 14.6 & 7.0\\ 5.0 & 35.8 & 4.2\\ 2.0 & 8.0 & 29.0 \end{array}\right]$
				0.80±0.03	0.69±0.05	
				0.74±0.04	0.86±0.03	

**Table 7:** Classification performance of the multimodal speech feature set (CHMLTB) across different fusion and reorganization strategies (metrics for AD/HC/MCI are stacked vertically).

Feature	Dim	Accuracy	Sensitivity (AD/HC/MCI)	Specificity (AD/HC/MCI)	Confusion Matrix
CHMLTB	294	0.8202±0.0041	0.8857±0.0000	0.8333±0.0000	$\left[\begin{array}{ccc} 31.0 & 0.0 & 4.0\\ 6.0 & 35.6 & 3.4\\ 8.0 & 0.0 & 31.0 \end{array}\right]$
			0.7911±0.0109	1.0000±0.0000	
			0.7949±0.0000	0.9075±0.0061	
CHMLTB-256	256	0.8286±0.0086	0.8629±0.0214	0.8500±0.0058	$\left[\begin{array}{ccc} 30.2 & 0.6 & 4.2\\ 4.8 & 37.2 & 3.0\\ 7.8 & 0.0 & 31.2 \end{array}\right]$
			0.8267±0.0259	0.9919±0.0066	
			0.8000±0.0103	0.9100±0.0146	
**CHMLTB-128**	**128**	0.8286±0.0041	0.8743±0.0140	0.8429±0.0048	$\left[\begin{array}{ccc} 30.6 & 0.6 & 3.8\\ 5.6 & 36.6 & 2.8\\ 7.6 & 0 & 31.4 \end{array}\right]$
			0.8133±0.0109	0.9919±0.0066	
			0.8051±0.0126	0.9175±0.0100	
CHMLTB-64	64	0.8218±0.0034	0.8800±0.0114	0.8333±0.0000	$\left[\begin{array}{ccc} 30.8 & 0.0 & 4.2\\ 6.0 & 36.0 & 3.0\\ 8.0 & 0.0 & 31.0 \end{array}\right]$
			0.8000±0.0000	1.0000±0.0000	
			0.7949±0.0000	0.9100±0.0050	

**Table 8:** Alignment performance across different feature dimensions and common subspace capacities (metrics for AD/HC/MCI are stacked vertically).

EEG Set (Dim)	Speech Set (Dim)	Subspace Dim	Accuracy	Sensitivity (AD/HC/MCI)	Specificity (AD/HC/MCI)	Confusion Matrix
TFDMPI (2335)	CHMLTB (294)	128	0.8353±0.0067	0.8857±0.0000	0.8571±0.0151	$\left[\begin{array}{ccc} 31.0 & 0.0 & 4.0\\ 5.6 & 35.8 & 3.6\\ 6.4 & 0.0 & 32.6 \end{array}\right]$
				0.7956±0.0089	1.0000±0.0000	
				0.8359±0.0205	0.9050±0.0150	
TFDMPI-32 (32)	CHMLTB (294)	128	0.8336±0.0063	0.8914±0.0114	0.8476±0.0089	$\left[\begin{array}{ccc} 31.2 & 0.0 & 3.8\\ 5.6 & 36.2 & 3.2\\ 7.2 & 0.0 & 31.8 \end{array}\right]$
				0.8044±0.0089	1.0000±0.0000	
				0.8154±0.0103	0.9125±0.0079	
TFDMPI-32 (32)	CHMLTB-128 (128)	256	0.8471±0.0034	0.8857±0.0000	0.8571±0.0000	$\left[\begin{array}{ccc} 31.0 & 0.0 & 4.0\\ 4.0 & 38.8 & 2.2\\ 8.0 & 0.0 & 31.0 \end{array}\right]$
				0.8622±0.0089	1.0000±0.0000	
				0.7949±0.0000	0.9225±0.0050	
TFDMPI-32 (32)	CHMLTB-128 (128)	64	0.8588±0.0124	0.8400±0.0140	0.9048±0.0075	$\left[\begin{array}{ccc} 29.4 & 1.2 & 4.4\\ 1.2 & 40.8 & 3.0\\ 6.8 & 0.2 & 32.0 \end{array}\right]$
				0.9067±0.0259	0.9811±0.0066	
				0.8205±0.0000	0.9075±0.0170	
**TFDMPI-32** **(32)**	**CHMLTB-128** **(128)**	**128**	0.8689±0.0067	0.9029±0.0140	0.8857±0.0058	$\left[\begin{array}{ccc} 31.6 & 0.2 & 3.2\\ 3.0 & 40.0 & 2.0\\ 6.6 & 0.6 & 31.8 \end{array}\right]$
				0.8889±0.0000	0.9892±0.0101	
				0.8154±0.0192	0.9350±0.0050	

**Table 9:** Overall performance metrics of the ablation study.

Features	Model	Accuracy	Sens & Spec (AD/HC/MCI)	F1 Score	AUC
Align-CHMLTB	Align	0.8689±0.0067	Sens: 0.9029/0.8889/0.8154 Spec: 0.8857/0.9892/0.9350	0.8662±0.0070	0.9658±0.0019
Align-CHMLTB + Random-Noise	Align	0.7782±0.0909	Sens: 0.9943/0.8400/0.5128 Spec: 0.7000/0.9946/0.9900	0.7525±0.1306	0.9518±0.0049
CHMLTB-128 + Latent Representation	Flow	0.8672±0.0111	Sens: 0.9029/0.8933/0.8051 Spec: 0.9048/0.9649/0.9350	0.8645±0.0123	0.9003±0.0092
Latent Representation	Align + Flow	0.7210±0.0519	Sens: 0.5829/0.9333/0.6000 Spec: 0.8262/0.9730/0.7925	0.7022±0.0564	0.8767±0.0222
Align-CHMLTB + Latent Representation	Align+Flow	0.8773±0.0086	Sens: 0.8629/0.9378/0.8205 Spec: 0.9071/0.9730/0.9400	0.8726±0.0088	0.9711±0.0014
**Align-CHMLTB +** **Latent Representation**	**Align + Flow** **+ Attention**	0.8908±0.0053	**Sens: 0.8800/0.9556/0.8256** **Spec: 0.9190/0.9676/0.9525**	0.8862±0.0058	0.9153±0.0046

**Table 10:** Performance comparison with advanced methods on the same dataset.

Reference	Model	Accuracy (%)	Recall (%)	F1 (%)
Official baseline	IS10 + SVM	79.8	78.5	78.6
Yang et al. [43]	IS10 + BERT + TF-IDF + Neural Network	75.9	74.0	74.4
Zhang et al. [44]	Wav2vec2 XLSR-53 finetune + GRU	81.51	/	/
Qin et al. [26]	Wav2vec2.0 + Fully Connected Layer	83.2	82.8	82.8
**Proposed Method**	**Alignment Network + Rectified Flow**	**89.08**	**88.71**	**88.62**

**Table 11:** Demographic comparison between NCMMSC 2021 and Miltiadous et al.’s EEG dataset.

Characteristics	Speech Dataset: NCMMSC 2021	EEG Dataset: Miltiadous et al. [20]
Ethnicity	Chinese	European (implied non-Chinese)
Native Language	Mandarin Chinese	Non-Mandarin (Greek/English implied)
Sample Size	399 subjects (280 Train/119 Test)	65 subjects
Age (Mean ± SD)	Undisclosed	AD: 66.4 ± 7.9/HC: 67.9 ± 5.4
Sex	Public	Public
Cognitive Assessment	Official NCMMSC Diagnosis	MMSE (AD: 17.75 ± 4.5/HC: 30)

## Data Availability

The EEG data used in this study are derived from public domain resources. The dataset is available at https://openneuro.org/datasets/ds004504/versions/1.0.8, accessed on 11 September 2024.

## Abbreviations

The following abbreviations are used in this manuscript:

AD	Alzheimer’s Disease
ASR	Automatic Speech Recognition
AUC	Area Under the Curve
CAP	Corrected Automatic Speech Recognition Projection
CNN	Convolutional Neural Network
DWT	Discrete Wavelet Transform
FN	False Negative
FP	False Positive
GRL	Gradient Reverse Layer
HC	Healthy Controls
LLDs	Low-Level Descriptors
MAE	Masked Autoencoder
MCI	Mild Cognitive Impairment
Melspec	Mel-spectrograms
MLP	Multilayer Perceptron
MMSE	Mini-Mental State Examination
MSE	Multiscale Entropy
NCMMSC	National Conference on Man-Machine Speech Communication
ODE	Ordinary Differential Equation
PCA	Principal Component Analysis
PLI	Phase Lag Index
PNR	Pronoun-to-Noun Ratio
PSD	Power Spectral Density
RMS	Root Mean Square
ROC	Receiver Operating Characteristic
ROIs	Regions of Interest
SE	Squeeze-and-Excitation
SNR	Signal-to-Noise Ratio
STFT	Short-Time Fourier Transform
TF-IDF	Term Frequency–Inverse Document Frequency
TN	True Negative
TP	True Positive

## Appendix A

**Table A1:** Class-wise precision, sensitivity, specificity, and confusion matrix of the ablation study (metrics for AD/HC/MCI are stacked vertically).

Features	Model	Precision	Sensitivity (AD/HC/MCI)	Specificity (AD/HC/MCI)	Confusion Matrix
Align-CHMLTB	Align	0.8691±0.0071	0.9029±0.0140	0.8857±0.0058	$\left[\begin{array}{ccc} 31.6 & 0.2 & 3.2\\ 3.0 & 40.0 & 2.0\\ 6.6 & 0.6 & 31.8 \end{array}\right]$
			0.8889±0.0000	0.9892±0.0101	
			0.8154±0.0192	0.9350±0.0050	
Align-CHMLTB + Random-Noise	Align	0.7886±0.1565	0.9943±0.0114	0.7000±0.1371	$\left[\begin{array}{ccc} 34.8 & 0.0 & 0.2\\ 6.6 & 37.8 & 0.6\\ 18.6 & 0.4 & 20.0 \end{array}\right]$
			0.8400±0.0218	0.9946±0.0108	
			0.5128±0.2915	0.9900±0.0146	
CHMLTB-128 + Latent Representation	Flow	0.8661±0.0121	0.9029±0.0229	0.9048±0.0184	$\left[\begin{array}{ccc} 31.6 & 0.0 & 3.4\\ 3.0 & 40.2 & 1.8\\ 5.0 & 2.6 & 31.4 \end{array}\right]$
			0.8933±0.0218	0.9649±0.0220	
			0.8051±0.0384	0.9350±0.0094	
Latent Representation	Align + Flow	0.7119±0.0605	0.5829±0.0736	0.8262±0.0896	$\left[\begin{array}{ccc} 20.4 & 1.0 & 13.6\\ 0.0 & 42.0 & 3.0\\ 14.6 & 1.0 & 23.4 \end{array}\right]$
			0.9333±0.0000	0.9730±0.0000	
			0.6000±0.1930	0.7925±0.0322	
Align-CHMLTB + Latent Representation	Align + Flow	0.8734±0.0094	0.8629±0.0280	0.9071±0.0089	$\left[\begin{array}{ccc} 30.2 & 1.0 & 3.8\\ 1.8 & 42.2 & 1.0\\ 6.0 & 1.0 & 32.0 \end{array}\right]$
			0.9378±0.0089	0.9730±0.0000	
			0.8205±0.0229	0.9400±0.0122	
**Align-CHMLTB +** **Latent Representation**	**Align + Flow** **+ Attention**	0.8877±0.0062	0.8800±0.0280	0.9190±0.0089	$\left[\begin{array}{ccc} 30.8 & 1.4 & 2.8\\ 1.0 & 43.0 & 1.0\\ 5.8 & 1.0 & 32.2 \end{array}\right]$
			0.9556±0.0000	0.9676±0.0066	
			0.8256±0.0192	0.9525±0.0166	
